# Influence of Burn Severity on Avifaunal Communities in Southern Tropical Dry Deciduous Forest, Western Ghats

**DOI:** 10.1002/ece3.74098

**Published:** 2026-08-19

**Authors:** Rajagopal James Pradeeshwar, Kumar Parthasarathy Ramesh, Tharmalingam Ramesh, Riddhika Kalle

**Affiliations:** ^1^ Master's Program in Wildlife Science (Ornithology) Sálim Ali Centre for Ornithology and Natural History Coimbatore Tamil Nadu India; ^2^ Central University of Tamil Nadu Thiruvarur Tamil Nadu India; ^3^ The Conservator of Forests (Project Tiger), Karnataka Forest Department Aranya Bhawan, Ashok Puram Mysuru Karnataka India; ^4^ Division of Conservation Ecology Sálim Ali Centre for Ornithology and Natural History Coimbatore Tamil Nadu India; ^5^ Centre for Functional Biodiversity, School of Life Sciences University of KwaZulu Natal Pietermaritzburg South Africa; ^6^ Division of Environmental Impact Assessment Sálim Ali Centre for Ornithology and Natural History Coimbatore Tamil Nadu India

**Keywords:** burn severity, community ecology, ornithology, pyrodiversity, Western Ghats, wildfire

## Abstract

Climate change has exacerbated the extent and severity of forest fire in biodiversity hotspots. The variation in fire regime attributes creates diverse successional stages and structures, thus offering ecological niches for species occupancy and increasing the biodiversity, thereby supporting the pyrodiversity‐biodiversity hypothesis. Understanding the effects of postfire burn severity and landscape heterogeneity on bird communities is essential for developing forest fire mitigation measures in tropical protected areas. We systematically surveyed birds in 120 point counts, 5 years postfire, to represent burn severity classes (unburned, low, moderate, and high severity). We assessed the influence of burn severity (a single‐fire component of pyrodiversity) on bird communities in a southern tropical dry deciduous forest of Bandipur Tiger Reserve, Nilgiri Biosphere Reserve in the Western Ghats. We calculated delta normalized burn ratio (dNBR) to classify burn severity using Landsat data. Richness, evenness, and Simpson diversity were not significant across burn severity. While Shannon diversity was marginally significant before testing the Bonferroni's correction, no individual burn severity class showed a significant difference in Shannon diversity after applying the correction. Bird community composition was significantly dissimilar between all burn severity classes (F = 4.52, *p* = 0.001). Among the foraging substrate guild, abundance of ground and generalist species was significantly higher in high burn severity than that of unburned. We found a significant positive relationship between burn severity (dNBR) and abundance (*β* = 1.41, *p* = 0.009) corroborating with the pyrodiversity‐biodiversity hypothesis. Forest fire management should account for the landscape heterogeneity created by fire. Implementing prescribed fires in highly vulnerable areas reduces fuel loads and prevents large, high burn severity areas within the reserve. In tropical environments, this study establishes a foundational understanding of the pyrodiversity‐avian diversity relationship, underscoring the necessity for future investigations employing multi‐taxa and multi‐scale approaches to discern underlying patterns for improving forest fire management.

## Graphical abstract

Effects of burn severity [pyrodiversity] on avian community were studied in a tropical deciduous forest of Western Ghats. Bird community composition varied significantly across burn severity classes and the pyrodiversity‐biodiversity hypothesis was supported. Functional guilds showed contrasting patterns in the postfire environment. Prescribed fires are a mitigation strategy to reduce high severity burns, while the latter tends to change the forest communities to more shrubland and grassland type.

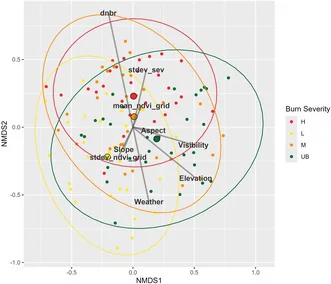

## 1 Introduction

In the Pyrocene, there is an increase in the frequency, severity, and extent of wildfires as a result of climate change which can influence the local flora and fauna (Westerling et al. 2006; He et al. 2019; Nimmo et al. 2021; Pyne 2021; Jones et al. 2023; Driscoll et al. 2024). While fire is a fundamental ecological process that has historically shaped the distribution of ecosystems, landscape patterns, and biodiversity globally (Turner et al. 2010; He et al. 2019; McLauchlan et al. 2020; Pausas et al. 2025), across all fire‐prone ecosystems, fire influences nutrient cycling, resets succession, and creates novel ecological niches for the species to occupy (Bond and Keeley 2005). Fire regimes are the collection of (i) temporal (fire frequency), (ii) spatial (burned patch size), and (iii) non‐spatiotemporal (severity) fire attributes in a landscape. Fire regime, whether historical or contemporary, results in distinct levels of pyrodiversity due to indigenous stewardship (Trauernicht et al. 2015; Hoffman et al. 2021). Pyrodiversity is quantified by the variation in multiple fire regime attributes. Martin and Sapsis (1992) hypothesized that variation in fire regime attributes (pyrodiversity) would create diverse successional stages and structures, thus offering ecological niches for species to occupy, thereby increasing biodiversity (i.e., pyrodiversity begets biodiversity). Pyrodiversity is often an important driver of biodiversity, though it has been largely overlooked until relatively recently (Jones and Tingley 2022). A global review by Kelly et al. (2020) found that 4403 (15%) out of 29,304 terrestrial and freshwater species are threatened by the change in fire regime. To advance fire management planning in protected areas, understanding how fire alters the species assemblage and composition is essential for developing effective mitigation measures. Birds are recognized as robust indicators of ecosystem integrity, owing to their ubiquitous presence (Carignan and Villard 2002), and can be useful model taxa for testing burn severity effects on community patterns (Beal‐Neves et al. 2020). Their trophic diversity, high detectability, and sensitivity to the vegetation structure are characteristics that make them vulnerable to fire‐mediated disturbances (Rainsford et al. 2023; Scott and Korb 2024). Bird species respond differently, owing to their life history and evolution (Canterbury et al. 2000). Research to date has covered various aspects in which pyrodiversity has been quantified and compared with the taxa of interest, thereby increasing our understanding of how pyrodiversity influences biodiversity in different ecosystems and taxa. Barlow and Peres (2004) sampled birds up to 3 years after widespread surface fire, and found that bird communities are becoming increasingly dissimilar from that of unburned plots. Later, numerous studies focused on how pyrodiversity affects alpha (Taylor et al. 2012; Bell et al. 2026) and beta diversity (Pastro et al. 2011; Leavesley and Cary 2013; Farnsworth et al. 2014; Bell et al. 2026). Few studies measured the variation in single‐fire attributes, such as severity (Tingley et al. 2016; Steel et al. 2019) or frequency (Taylor et al. 2012; Brown and York 2017; Coelho et al. 2023). Additionally, studies focused on species‐specific responses to postfire conditions in birds (Hutto et al. 2008; Tingley et al. 2020; Stillman et al. 2021). Mechanisms such as habitat complementation, habitat heterogeneity, and habitat refuge can give rise to the observed pyrodiversity‐biodiversity relationships (Jones and Tingley 2022).

In India, majority of the studies are focused on how flora is affected by forest fire such as reduced species diversity and regeneration in plants (Kodandapani et al. 2008). Similarly, higher tree species diversity was observed at moderate frequency, but decreased with increasing fire frequency, with fire‐tolerant species becoming more dominant (Ray et al. 2023). Fire‐affected areas having greater stem density, had lower species diversity due to increased dominance of fire‐tolerant species (Sukumar et al. 2005; Verma and Jayakumar 2015; Neeraja et al. 2021). However, the long‐term recovery may be impacted more by burn severity than by vegetation type and structure or by postfire climatic conditions (Torres et al. 2018) which can convert forests and woodlands into scrublands and grasslands (Cochrane 2003; Barlow and Peres 2004). Subsequently, frequently burned forest might also be more vulnerable to invasion by invasive fire‐adapted species, such as *Lantana camara*, *Prosopis juliflora* , and *Parthenium hysterophorus* (Hiremath and Sundaram 2005) as is the case in several protected areas in India. As a result, it creates a positive feedback loop of *Lantana camara* invasion along with the fire (fire‐Lantana cycle) (Hiremath and Sundaram 2005). There is evidence that environmental heterogeneity broadly drives variation in species richness (Stein et al. 2014). This variation in bird communities is majorly influenced by the geographical location, vegetation structure at a regional‐scale, and floristics at a local‐scale, altered by fire (Wiens and Rotenberry 1981; Martin and Sapsis 1992; Jayapal et al. 2009). Although areas that have been burnt and those that have not, often exhibited comparable levels of species richness and diversity, they commonly show pronounced dissimilarities in community composition as a result of species turnover (Barlow and Peres 2004; Beal‐Neves et al. 2020; Scott and Korb 2024).

Species were categorized into functional guilds based on life history strategies such as foraging substrate and foraging technique. Postfire habitat provides diverse niches for the species. High severity areas typically harbor insectivores and generalist species (Hutto and Patterson 2016). In addition to that, growth of shrubs following low and moderate fire severity favors shrub‐dwelling species (Latif et al. 2022). Therefore, assessing functional guilds helps identify resource availability in postfire habitats and bird community compositions.

Studies testing pyrodiversity‐biodiversity relationships are known from sub‐tropics and temperate regions (Jones and Tingley 2022). To our knowledge there is no study that has tested relationships between burn severity (a single‐fire component of pyrodiversity) and bird community structure and composition in Asia. Therefore, our study tried to address this knowledge gap by understanding how burn severity (a single‐fire component of pyrodiversity) influences avian diversity (biodiversity) in tropical ecosystem, specifically in southern tropical dry deciduous forests of the Western Ghats, a global biodiversity hotspot using birds as an indicator taxon. The objectives were as follows: (i) to determine how avian richness, diversity, and evenness vary among burn severities (unburned, low, moderate, and high); (ii) to assess the variation in avian community composition and functional guild across different burn severities and (iii) to test the pyrodiversity‐biodiversity hypothesis by treating burn severity as a single‐fire, single‐attribute of pyrodiversity framework i.e., does bird abundance increase with increasing burn severity.

## 2 Methods

### 2.1 Study Area

**FIGURE 1:**
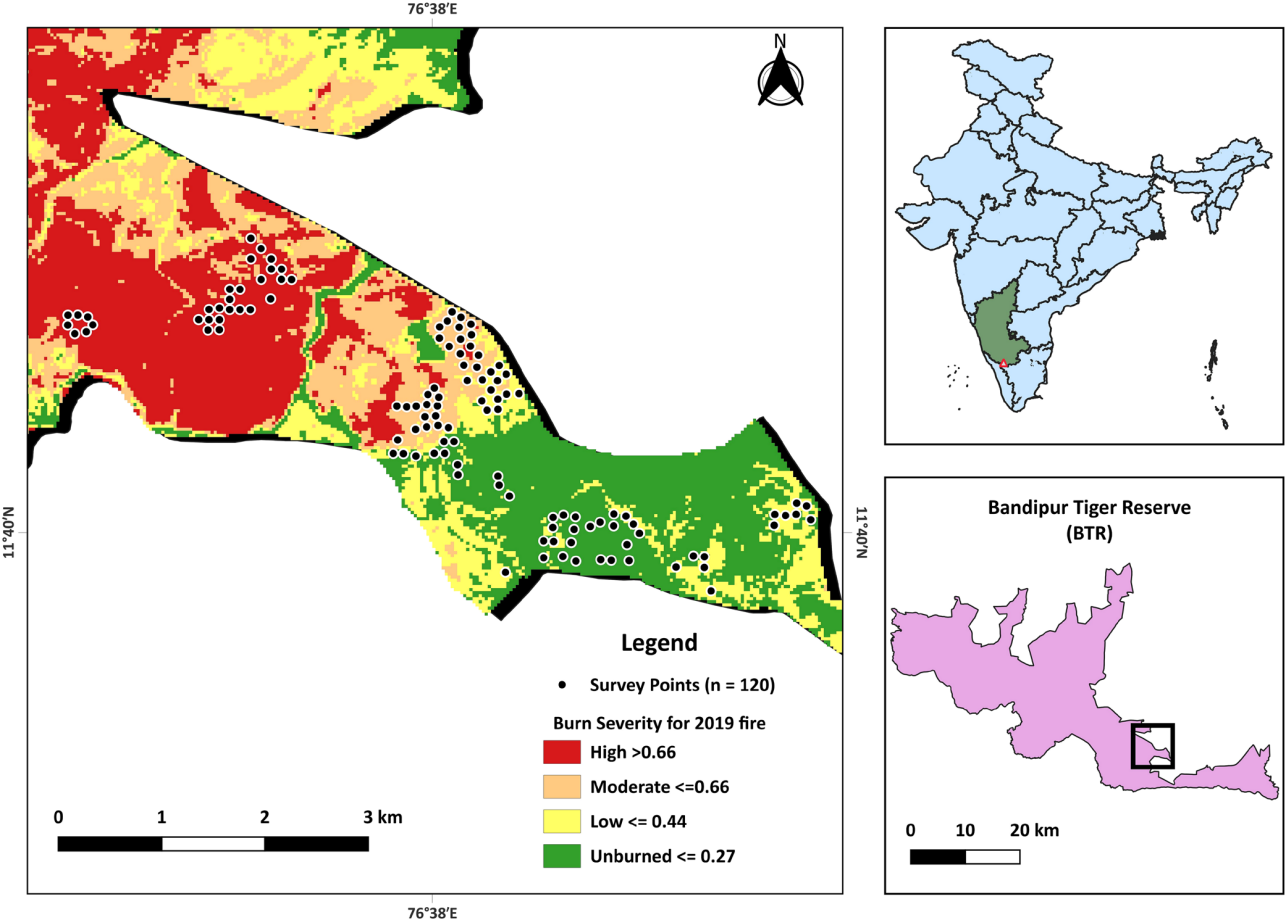
Study area map of 2019 forest fire in Bandipur Tiger Reserve, Karnataka, India. Burn severity classified using modified delta Normalized Burn Ratio.

Bandipur Tiger Reserve (BTR) spans parts of Mysore and Chamarajanagar districts in southern Karnataka, India. Encompassing 1456.309 km^2^, BTR forms a critical component of the Nilgiri Biosphere Reserve and is ecologically contiguous with Nagarahole Tiger Reserve to the northwest, Mudumalai Tiger Reserve to the south, and Wayanad Wildlife Sanctuary to the southwest. Geographically, the reserve extends between latitudes 11°35′34″ N and 11°55′02″ N to longitudes 76°12′17″ E and 76°51′32″ E (Figure 1). Administratively, BTR is organized into 11 ranges: Gundre, N. Begur, Moliyur, A.M. Gudi, Hediyala, Moolehole, Maddur, Omkara, G.S. Betta, Bandipur, and Kundakere, with elevations ranging from 680 m to 1454 m above sea level. The region experiences a tropical climate marked by distinct wet (June–September) and dry (January–May) seasons. Annual temperatures fluctuate between 10°C and 35°C, with mean precipitation of 1200 mm. The prolonged dry season provides ideal conditions for forest fires as temperatures rise upto 35°C. These conditions combined with high availability of dry leaf‐litter and grasses on the forest floor along with high wind speed provides suitable conditions for fire ignition and rapid spatial spread of the fire. Vegetation is predominantly characterized by dry deciduous scrub (scrub forest) in the eastern part of the reserve, southern tropical dry deciduous forests in the central part, and southern tropical moist deciduous forests in the western part, as classified by Champion and Seth (1968). BTR is classified within the intermediate‐cool‐small pyrome, which is characterized by intermediate fire return but fairly small fires (Archibald et al. 2013). Forest fires in BTR are exclusively surface fires (Ramesh 2024), with severity modulated by climatic conditions, vegetation structure, and topographical features. BTR has a history of recurrent massive fires, particularly severe episodes in 2012, 2017 and 2019 (Ananth et al. 2019). Recent decades have witnessed a progressive escalation in fire frequency and extent (Somashekar et al. 2009; Kumar et al. 2025), driven largely by anthropogenic activities such as non‐timber forest product (NTFP) collection, livestock grazing, poaching, tourism, and encroaching settlements (Srivastava et al. 2014). To mitigate the fire risks, prescribed burning is employed as a strategy for fuel load reduction (Ramesh 2024). Notably, the 2019 forest fire event coincided with an extreme drought period, resulting in heterogeneous burn severity patterns across the landscape (Ramesh 2024; Balakavi et al. 2025).

### 2.2 Fire Covariates (Burn Severity)

The most recent major forest fire event in BTR occurred in 2019, following a period of severe drought. Such large‐scale fires can have an impact on the local bird communities. To assess the extent and severity of the 2019 fire, Landsat 8 imagery with a spatial resolution of 30 m was used for both the pre‐ (January) and postfire (March) images of the study area from USGS Earth explorer (https://earthexplorer.usgs.gov/). All downloaded data were L1T and re‐projected to the UTM coordinate system (WGS 84, UTM, Zone 43 N). After preprocessing, we calculated the Normalized Burn Ratio (NBR), which is the normalized ratio between near‐infrared (NIR) and short‐wave infrared (SWIR) radiation (Equation 1). NIR and SWIR bands of satellite sensors respond in opposite ways to burned vegetation, allowing to identify burned areas (Key and Benson 2006). To obtain a quantitative measure of change, we calculated the delta Normalized Burn Ratio (dNBR) as a measure of vegetation change due to fire (García and Caselles 1991; Key and Benson 2006) (Equation 2).

(1) $$\mathrm{NBR}=\left(\mathrm{NIR}–\text{SWIR}\right)/\left(\mathrm{NIR}+\text{SWIR}\right)$$

(2) $$\text{dNBR}={\mathrm{NBR}}_{\text{Prefire}}–{\mathrm{NBR}}_{\text{Postfire}}$$

We modified the classification of the delta Normalized Burn Ratio (dNBR) to better suit the characteristics of southern tropical dry deciduous forest. The classification of dNBR is as follows: (a) unburned (dNBR < = 0.27); (b) low burn severity (dNBR < = 0.44); (c) moderate burn severity (dNBR < = 0.66); (d) high burn severity (dNBR > 0.66). In highly seasonal tropical dry deciduous forest, natural phenological changes create high variance in the vegetation density between the pre‐ and postfire data. By setting a higher threshold for unburned (≤ 0.27) we minimized the false positives caused by seasonal deciduous phenology. Although systematic field validation was not performed, the data was cross‐verified with the field forest staff for reasonable accuracy. Thus, the thresholds used in the classification of dNBR reasonably captured field observations.

### 2.3 Bird Sampling Design

We used burn severity attribute as a component of pyrodiversity to understand the influence on bird communities caused due to the 2019 forest‐fire and was sampled 5 years postfire. We only chose southern tropical dry deciduous forest type from the reclassified version of Land‐Use Land‐Cover 2015 (Roy et al. 2015). Five years postfire in a southern tropical dry deciduous forest represents an intermediate secondary successional stage which is characterized by rapid shrub recovery following fire, with herbaceous and early‐successional species often recolonizing within one to two seasons. At 5 years postfire, low severity areas are likely to have recovered understory structure, while moderate and high severity areas may still exhibit reduced and altered tree and shrub species assemblages. We then clipped the burn severity classes with the southern tropical dry deciduous forest type and overlaid 100 m x 100 m grids using QGIS 3.24 (QGIS Development Team 2025). We generated centroids for each grid to conduct bird surveys where each centroid had a point count station. Sampling effort was distributed equally across all burn severity classes (*n* = 30/burn severity class) (Figure S1). Bird communities were surveyed using 120 fixed‐width point counts of 50 m radius and each point count station was separated by a minimum distance of 100 m (Bibby et al. 2000). As the geographical area of the forest fire is small and fragmented in the tropical dry deciduous forest, thereby we maintained 100 m interpoint distance to capture the total species richness (Morrison and Peitz 2020). Bird surveys were conducted from May to August, with 15‐min point counts conducted during the morning hours (08:00–11:00 AM). Birds were identified to the species‐level in the field, both visually and by their vocalizations. We only recorded bird species that were detected within the 50 m radius of the point count. The number of individual birds was always noted down in the case of visual observations, and recorded as the minimum number of birds distinctly heard calling when birds were detected aurally. All point‐count stations were visited only once.

### 2.4 Environmental Covariates

In all the 120 point‐count stations, environmental variables were recorded to understand their influence on the avian diversity, which includes (i) elevation at 30 m resolution derived from Shuttle Radar Topography Mission (SRTM) digital elevation model; (ii) weather, recorded during the survey (six types—Sunny, Sunny + Windy, Cloudy, Cloudy + Windy, Cloudy + Drizzling, and Cloudy + Drizzling + Windy); (iii) visibility (very low, low, moderate, and high visibility), taken as a proxy for invasive species cover (cover of invasive plants such as *Lantana camara*, *Senna spectabilis* , *Chromolaena odorata* , and *Parthenium hysterophorus* from the point‐count station of each sampled plot). Very low visibility corresponds to the thick cover due to both native and invasive vegetation and high visibility corresponds to the open habitat with relatively less vegetation. We also calculated vegetation heterogeneity (as standard deviation of NDVI within each grid) and variation in burn severity (as standard deviation of burn severity (dNBR) within each grid) using the same Landsat data (Supplementary Table S1).

### 2.5 Data Analysis

A total of 12 bird species which had less than two detections were excluded from analysis as their low detections were insufficient to model habitat associations across burn severity classes, instead we focused on the breeding resident birds (Supplementary Table S2A). Richness (S), Shannon diversity (H), Simpson diversity (1–D), and Evenness (J) of the bird species across the burn severity classes was calculated using the vegan package (Oksanen et al. 2024). We determined differences using Kruskal–Wallis tests, followed by pairwise Dunn tests with Bonferroni corrections. We performed non‐metric multidimensional scaling (NMDS) to visualize the differences in community composition, using species abundance from each point‐count across burn severity classes using *metaMDS* function and used *envfit* function for the underlying environmental variables using the vegan package. We ran the NMDS ordination using Bray‐Curtis distance measure and then used permutational multivariate analysis of variance (PERMANOVA) with 999 permutations, using *adonis2* function in vegan package to compare avian community composition among burn severities (unburned, low, moderate, and high). We classified avian species into functional guild categories (foraging substrate—shrub‐lower canopy, ground, and generalist; foraging technique—probing species) adapted from De Graaf et al. (1985) and Cornell Birds of the World (Billerman et al. 2025) and compared their abundances across different classes (unburned, low, moderate, and high) of burn severity. To test the relationship between species abundance and predictor variables, we used linear model using base R function “*lm*” at point‐count level. Separate models were developed for total bird abundance and for the abundance of select foraging substrate guilds (ground, shrub‐lower, upper canopy, and generalist). These foraging substrate guilds were considered because they represent distinct habitat‐use patterns that are likely to be affected by postfire vegetation structure and burn severity. The predictor variables included burn severity (dNBR), weather, visibility, elevation, vegetation heterogeneity (std_ndvi_grid), and variation in burn severity (std_sev). All predictors were tested for multicollinearity using Pearson's correlation and only uncorrelated variables were used for further analysis (Figure S2). Models with less than or equal to two ΔAIC will be selected for interpretation. In case of multiple models ranked less than two ΔAIC, model averaging was performed. All statistical analyses were conducted in R version 4.4.1 (R Core Team 2025) with α = 0.05.

## 3 Results

### 3.1 Avian Community Composition Across Burn Severity Classes

**FIGURE 2:**
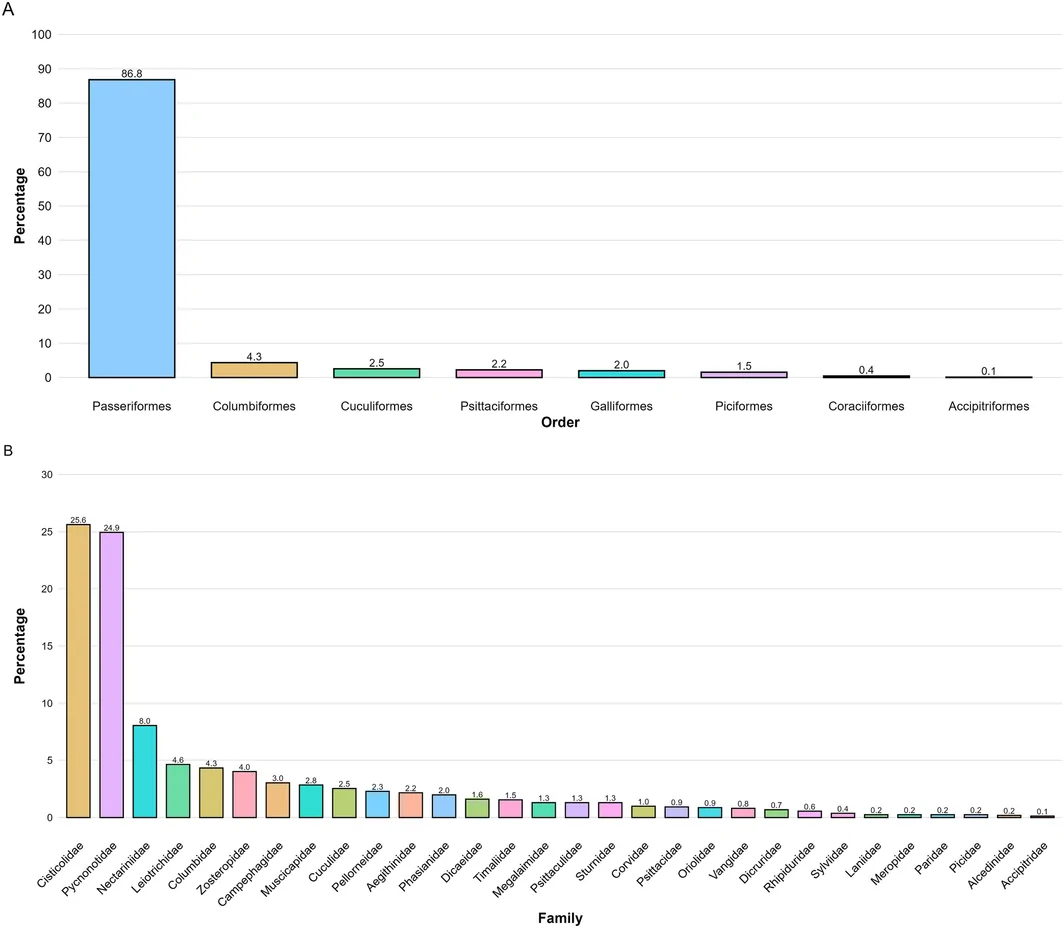
Percentage composition of bird (A) order and (B) families recorded across all burn severity classes within the study area.

**FIGURE 3:**
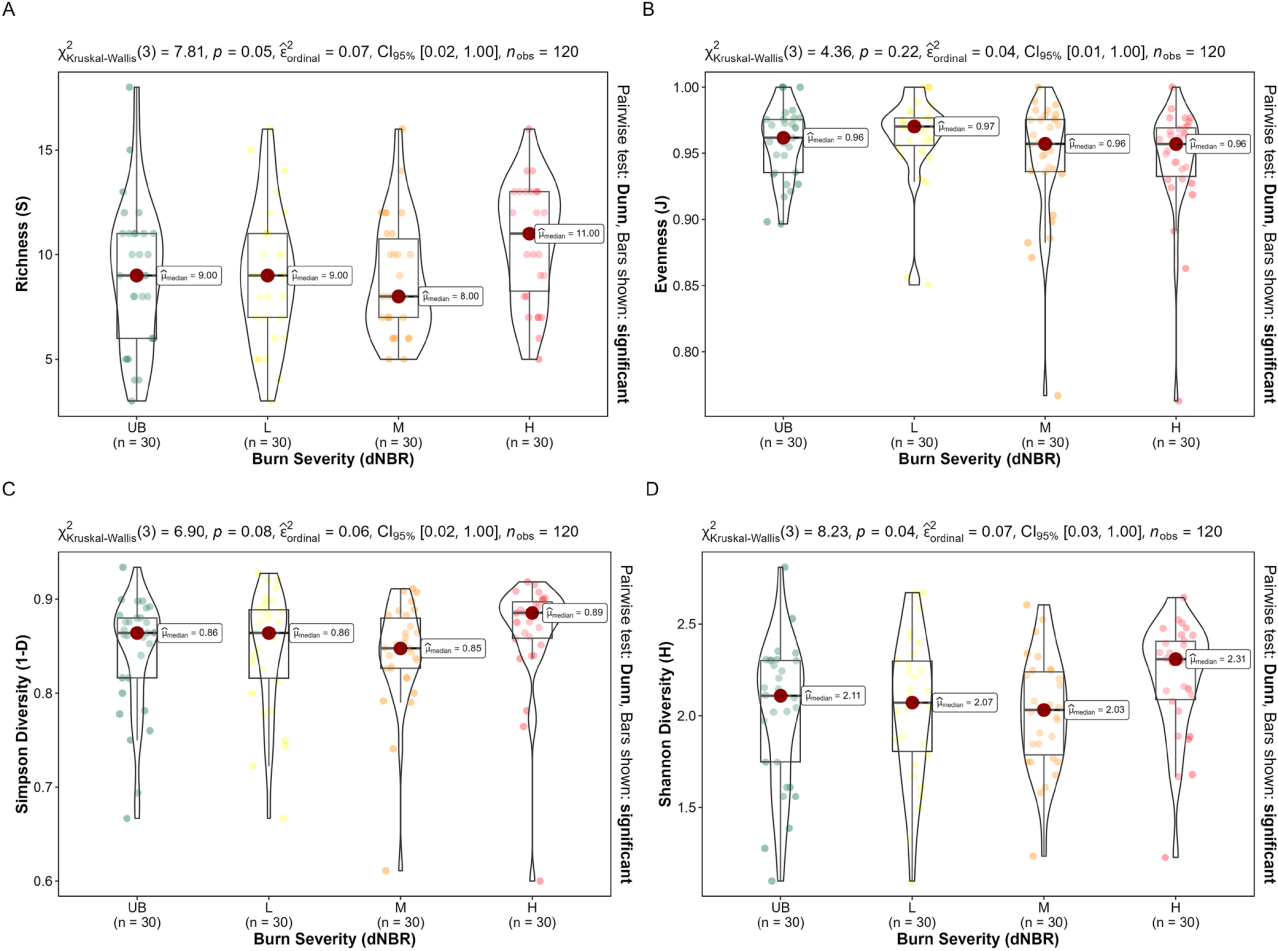
Variation in (A) species richness, (B) evenness, (C) Simpson diversity, (D) Shannon diversity across burn severity categories (unburned, low, moderate, and high) with the 2019 forest fire, 5 years postfire. Significant differences among burn severity were identified using the Kruskal‐Wallis test followed by pairwise Dunn test with Bonferroni correction.

Spatial autocorrelation analysis using Moran's I at 100 m distance threshold indicated no significant spatial autocorrelation in any of the abundance or diversity variables. Moran's I values ranged from 0.066 for Shannon diversity to 0.183 with *p* > 0.05. The observed Moran's I values were slightly positive compared with the expected value, suggesting weak positive spatial correlation, but statistically not significant. We recorded a total of 1616 individuals of 56 species, corresponding to 8 orders and 30 families (Supplementary Table S2B). While most species belonged to the Passeriformes order (86.8%) (Figure 2A), the Cisticolidae and Pycnonotidae were the most represented families, with 26% and 25% respectively across all burn severity classes (Figure 2B). Richness, Simpson diversity, and evenness did not show any significant difference across burn severity classes (*p* > 0.05) except for Shannon diversity (*p* < 0.05) (Figure 3A–D). The pairwise test using Bonferroni corrections found no significant difference in Shannon diversity (*p* > 0.05).

**FIGURE 4:**
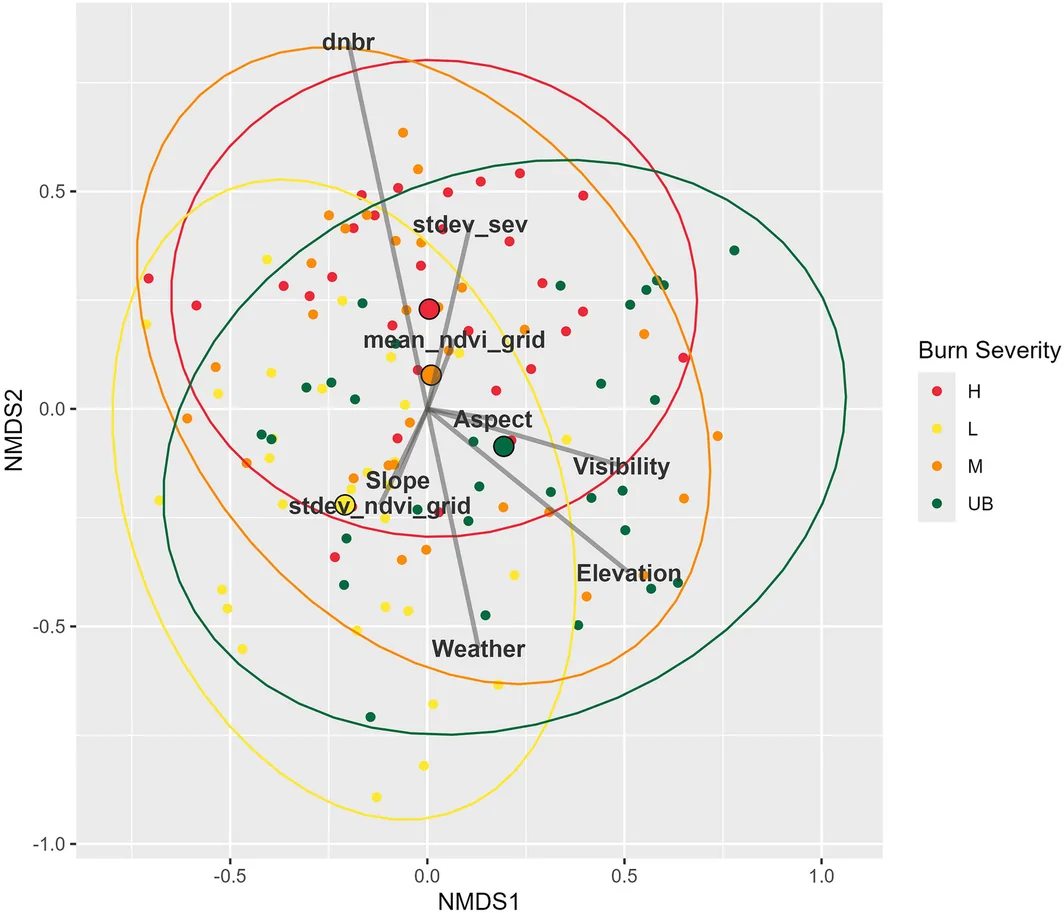
Non‐metric multidimensional scaling (NMDS) plot with envfit of avian community composition data by burn severity (Unburned—UB, low—L, moderate—M, and high—H) within the 2019 forest fire, 5 years postfire. Species assemblages were significantly different across burn severities, according to a Permanova analysis of variance (F = 4.5222, *p* = 0.001) with a stress = 0.17, *k* = 4, distance measure = Bray‐Curtis. Pairwise comparisons found significant differences between all burn severities (*p* < 0.01). Each dot represents a bird point count station (*n* = 120, 30 points per burn severity) and ellipses indicate 95% confidence interval of the plots by burn severity. The gray lines is the envfit vector which represent the influence of environmental variables on bird community.

Avian community assemblages in the 2019 forest fire were significantly different across burn severities, according to PERMANOVA (F = 4.52, *p* = 0.001), Stress = 0.17, k = 4, distance measure = Bray‐Curtis. Pairwise comparisons between groups revealed that all the pairs were significantly different in their community composition: high‐low (F = 8.252, *p* = 0.001); high‐moderate (F = 4.0552, *p* = 0.001); high‐unburned (F = 5.2209, *p* = 0.001); low‐moderate (F = 3.8829, *p* = 0.001); low‐unburned (F = 4.1976, *p* = 0.01); moderate‐unburned (F = 2.246, *p* = 0.008). The composition of avian community postfire was associated with dNBR, weather, visibility, and elevation (Figure 4).

### 3.2 Abundance of Bird Functional Guilds Across Burn Severity

**FIGURE 5:**
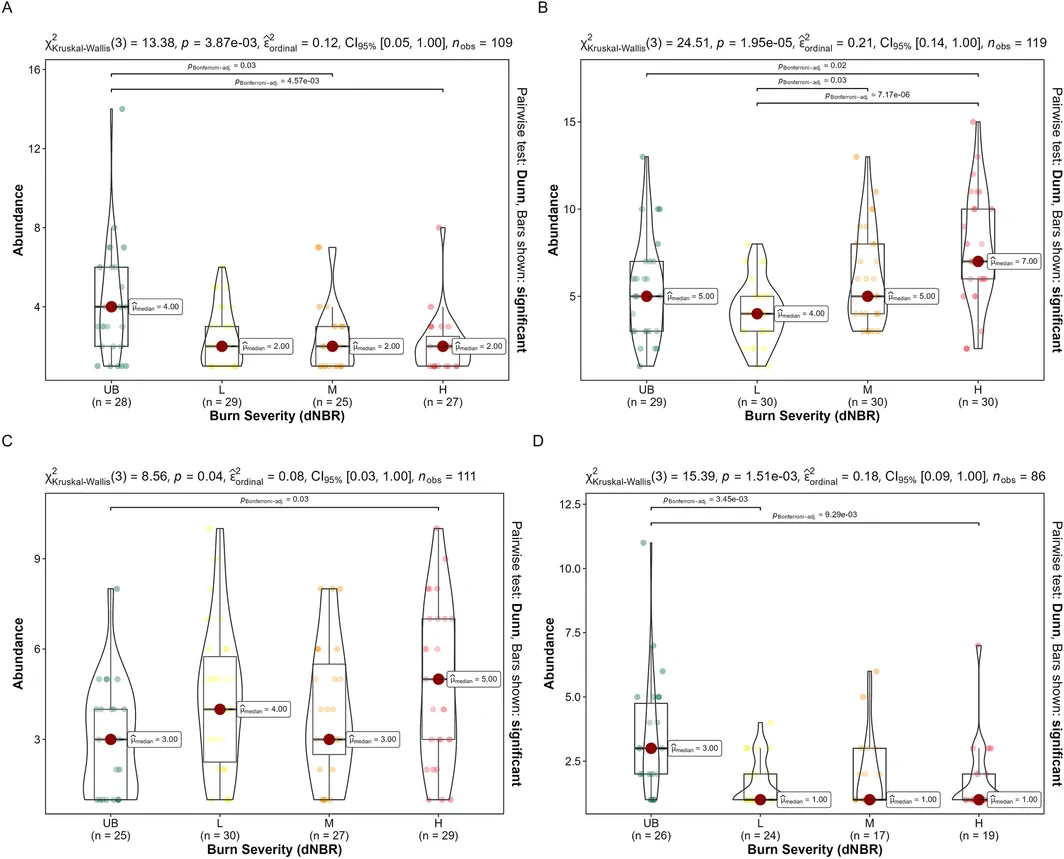
Variation in abundance of foraging substrate guild. (A) shrub‐lower canopy, (B) ground, (C) generalist species. In case of foraging technique guild (D) probing species across burn severity categories (unburned, low, moderate, and high) with the 2019 forest fire, 5 years postfire. Significant differences among burn severity were identified using the Kruskal‐Wallis test followed by pairwise Dunn test with Bonferroni correction.

Among the feeding substrates, we found equal proportion of generalists in high and moderate burn severity classes while the proportion of ground‐vegetation feeders was highest in low burn severity class. The proportion of shrub‐lower and upper canopy feeders showed similar responses peaking at unburned areas (Figure S3A). Among the foraging technique the proportion of probing species increased significantly in unburned areas while foragers declined in unburned areas, gleaners increased in high and moderate burn severity classes (Figure S3B). The mean abundance per burn severity class (Table S2B) shows that the high severity class had notably higher abundance followed by moderate and other categories. The most pronounced pattern observed was the marked difference in the abundance of functional guilds between unburned areas and those subjected to high burn severity. In foraging substrate category, shrub‐lower canopy foraging species were significantly abundant in in unburned area than high and moderate burn severity area (*p* < 0.05) (Figure 5A) whereas, ground foraging species were significantly abundant in high burn severity area compared with other burn severity classes including those of unburned (*p* < 0.05) (Figure 5B); foraging substrate generalist species were significantly abundant in high burn severity area than unburned area (*p* < 0.05) (Figure 5C). In foraging technique category, probing species were significantly abundant in unburned area than high burn severity area (*p* < 0.05) (Figure 5D).

### 3.3 Drivers of Total Avian Abundance

**FIGURE 6:**
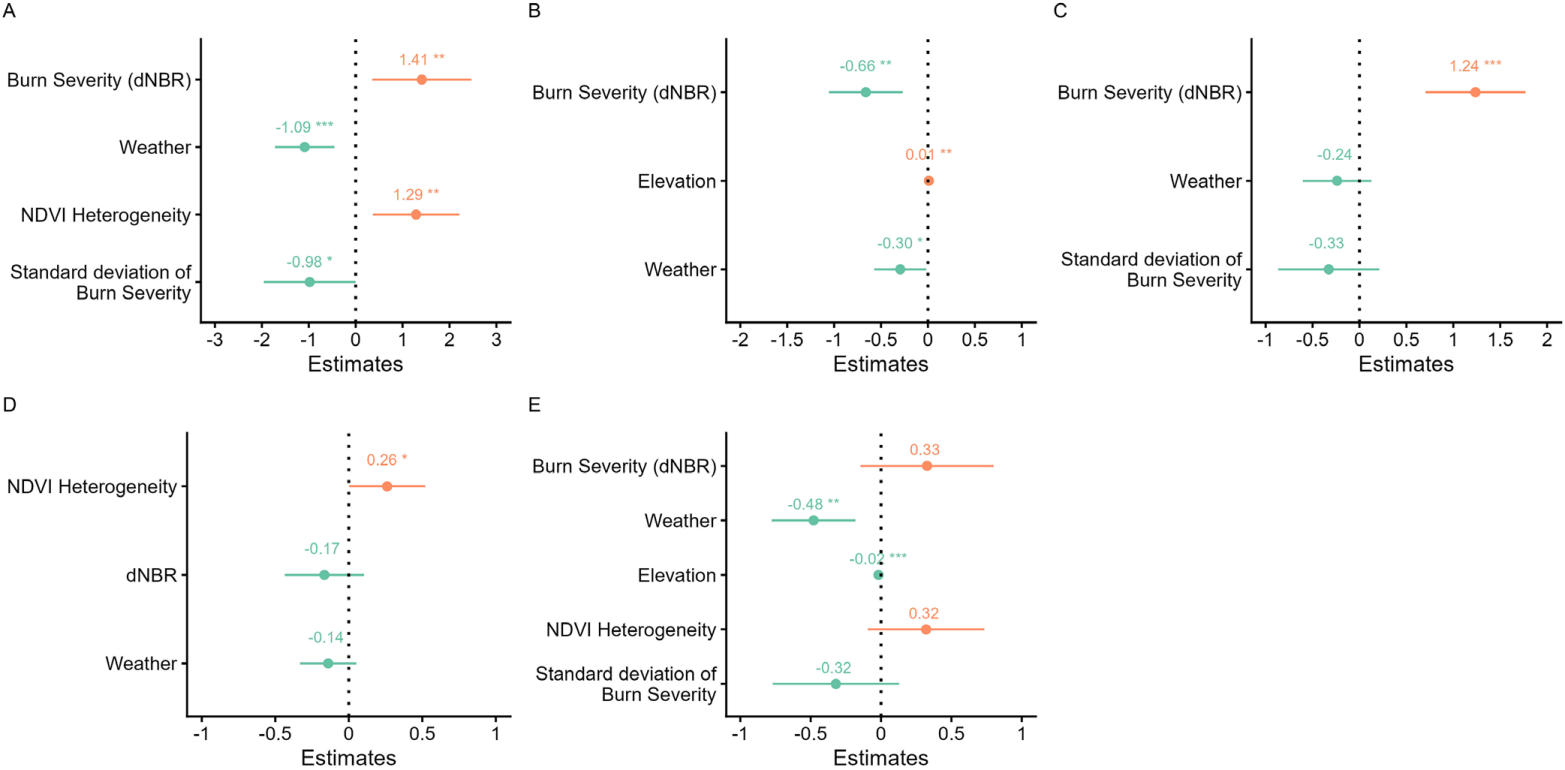
Beta estimates of the predictors of abundance of (A) total species, (B) shrub‐lower canopy foraging, (C) ground foraging, (D) upper canopy foraging, and (E) foraging substrate generalist species.

**FIGURE 7:**
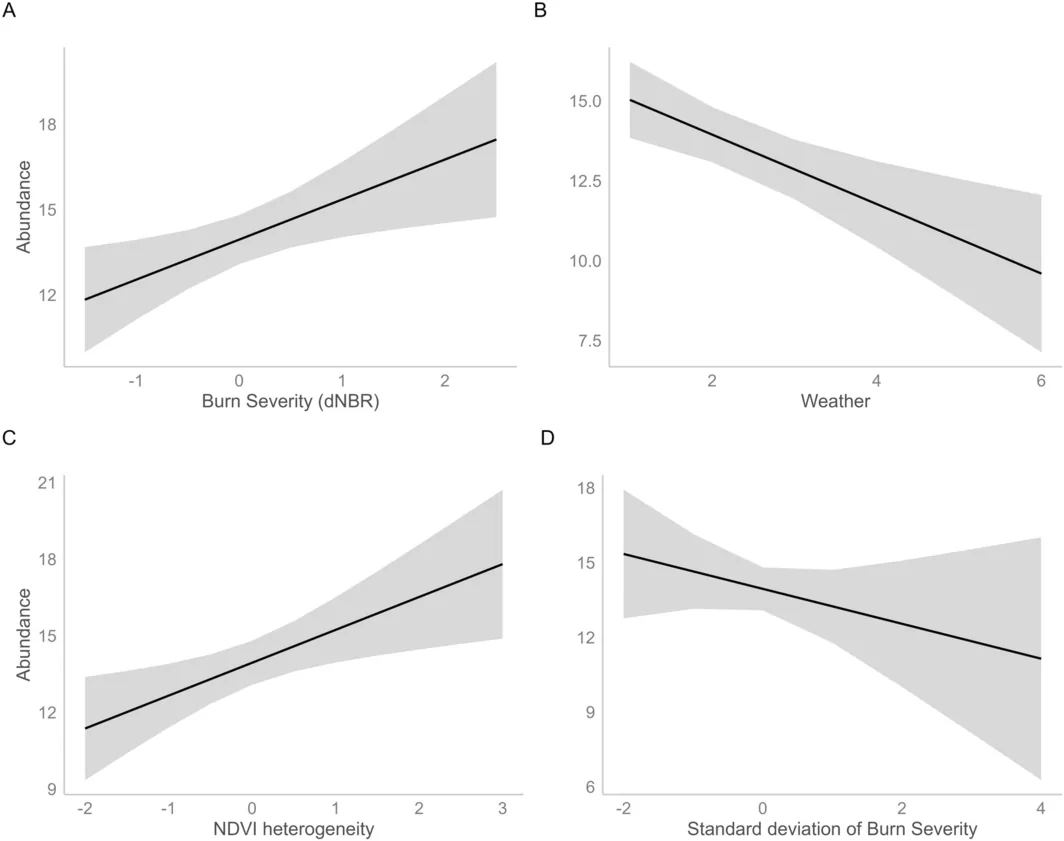
Model predicted relationships between total abundance and predictors: (A) burn severity (dNBR), (B) weather, (C) NDVI heterogeneity, and (D) standard deviation of burn severity. Gray shaded areas denote 95% confidence intervals.

Two models were less than two delta AIC with a total weight of 0.92 (Supplementary Table S3). These two models were then averaged to identify the key predictors, revealing the total bird abundance was associated with burn severity (dNBR), weather, vegetation heterogeneity (stdev_ndvi_grid), and heterogeneity in burn severity (stdev_sev) (Figures 6A and 7A–D). The averaged model explained a proportion of the variance in total bird abundance (R^2^ = 0.216). Burn severity (dNBR) had a significant positive effect on bird abundance (*β* = 1.41, 95% CI = 0.35, 2.47, *p* = 0.009), and vegetation heterogeneity (standard deviation of NDVI within the grid) also showed a positive relationship (*β* = 1.29, 95% CI = 0.37, 2.21, *p* = 0.006). In contrast, weather (*β* = −1.09, 95% CI = −1.72, −0.45, *p* = 0.001) and heterogeneity in burn severity (*β* = −0.98, 95% CI = −1.96, −0.00, *p* = 0.049) had a significant negative effect on the abundance.

### 3.4 Drivers of Foraging‐Guild Specific Avian Abundance

#### 3.4.1 Shrub‐Lower Canopy Foraging Species

**FIGURE 8:**
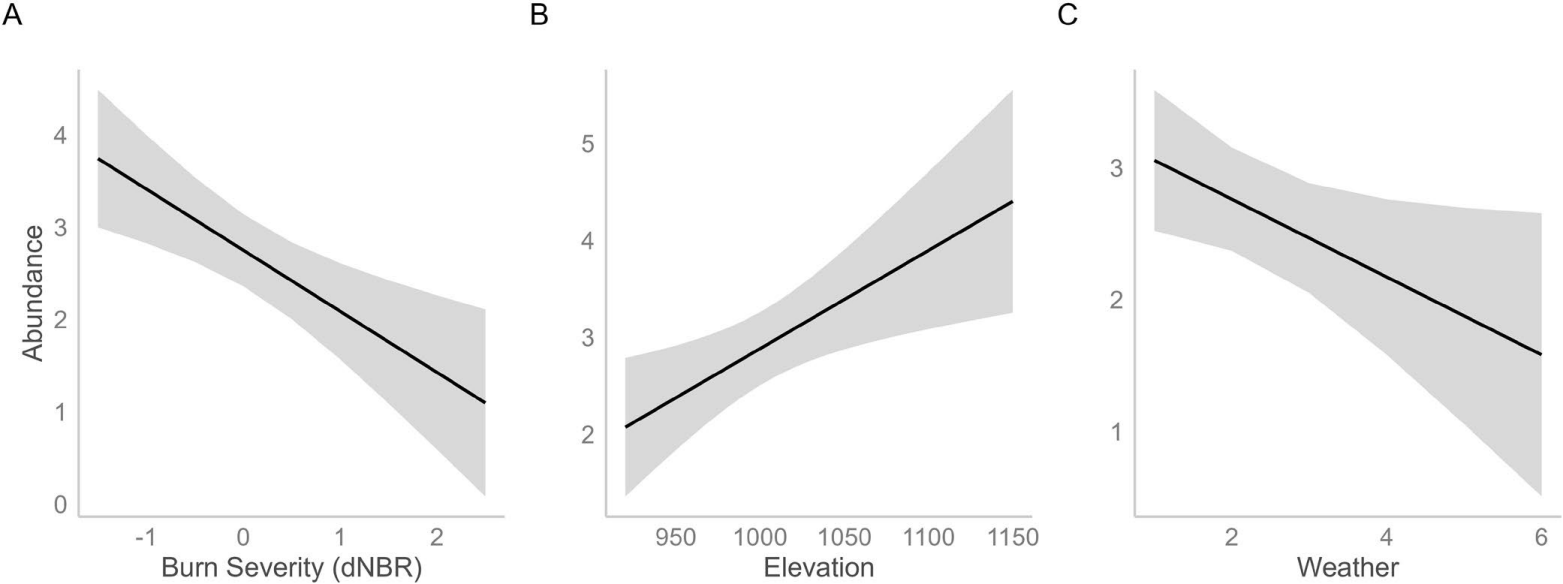
Model predicted relationships between abundance of shrub‐lower canopy foraging species and predictors: (A) burn severity (dNBR), (B) elevation, and (C) weather. Gray shaded areas denote 95% confidence intervals.

Based on the ranking, only one model was with less than two delta AIC with a weight of 0.65 (Table S4). This top model revealed that abundance of shrub‐lower canopy foraging species was associated with burn severity (dNBR), elevation, and weather (Figures 6B and 8A–D). The top model explained a proportion of the variance in abundance (F (3, 105) = 8.06, *p* < 0.001, R^2^ = 0.19). Burn severity (dNBR) (*β* = −0.66, 95% CI [−1.06, −0.27], *p* = 0.001) and weather (*β* = −0.30, 95% CI [−0.57, −0.02], *p* = 0.037) had significant negative effects on abundance of shrub‐lower canopy foraging species. On the other hand, elevation showed a small but significant positive effect (*β* = 0.01, 95% CI [0.00, 0.02], *p* = 0.007).

#### 3.4.2 Ground Foraging Species

**FIGURE 9:**
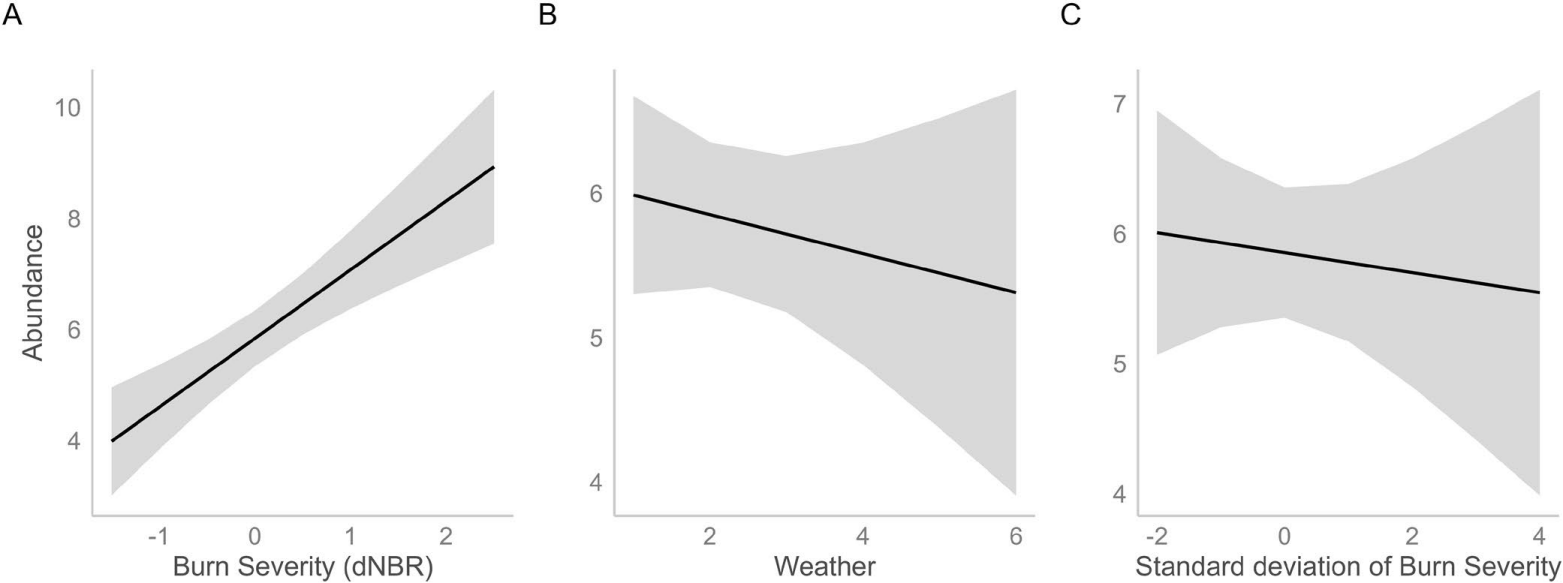
Model predicted relationships between abundance of ground foraging species and predictors: (A) burn severity (dNBR), (B) weather, and (C) standard deviation of burn severity. Gray shaded areas denote 95% confidence intervals.

Three models were less than two delta AIC with a total weight of 0.92 (Supplementary Table S5). These three models were then averaged to identify the key predictors, revealing the abundance of ground foraging species was associated with burn severity (dNBR), weather, and heterogeneity in burn severity (stdev_sev) (Figures 6C and 9A–D). The averaged model explained a proportion of the variance in total bird abundance of ground foraging species (R^2^ = 0.19). Burn severity (dNBR) had a significant positive effect on abundance of ground foraging species (*β* = 1.24, 95% CI [0.70, 1.77], *p* = < 0.001). By contrast, weather (*β* = −0.24, 95% CI [−0.60, 0.13], *p* = 0.201) and variation in burn severity (*β* = −0.33, 95% CI [−0.87, 0.21], *p* = 0.234) were negatively associated with bird abundance but not significantly.

#### 3.4.3 Upper Canopy Foraging Species

**FIGURE 10:**
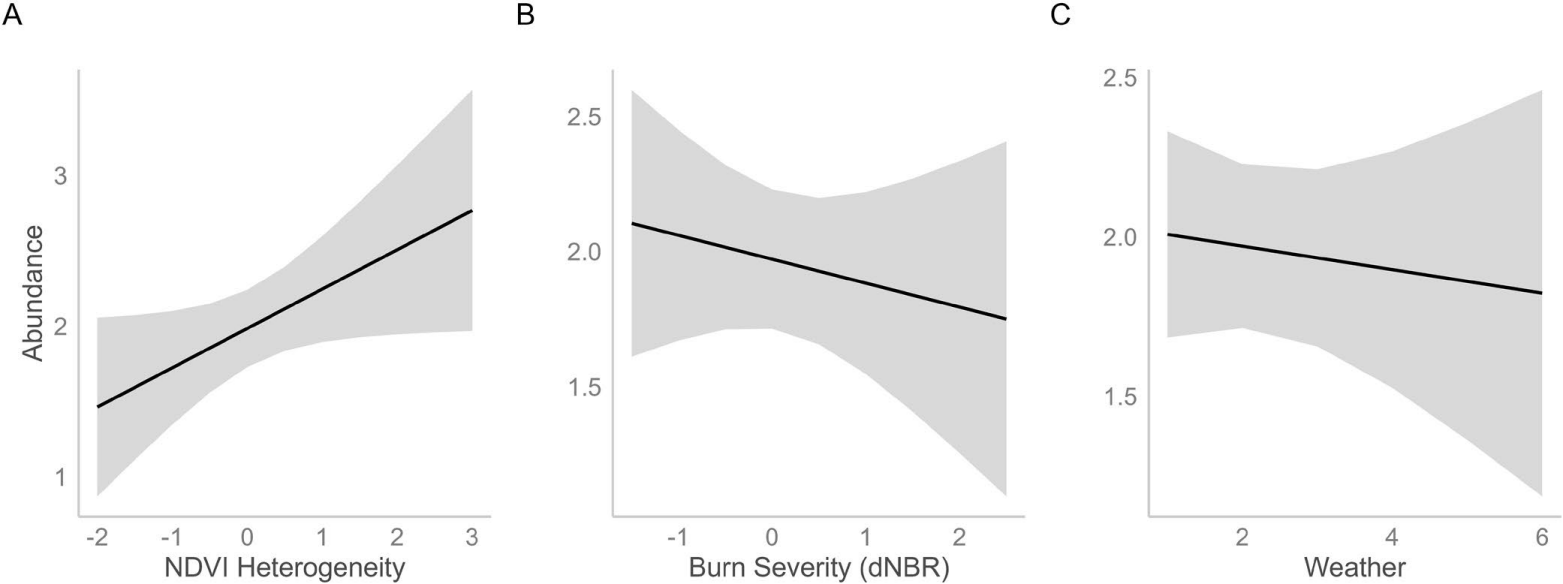
Model predicted relationships between abundance of upper canopy foraging species and predictors: (A) NDVI heterogeneity, (B) burn severity (dNBR), and (C) weather. Gray shaded areas denote 95% confidence intervals.

Three models were less than two delta AIC with a total weight of 0.63 (Supplementary Table S6). These three models were then averaged to identify the key predictors, revealing the abundance of upper canopy foraging species was associated with vegetation heterogeneity, burn severity (dNBR), and weather (Figures 6D and 10A–D). The averaged model explained a modest proportion of the variance in abundance of upper canopy foraging species (R^2^ = 0.069). Vegetation heterogeneity had a significant positive effect (*β* = 0.26, 95% CI [0.00, 0.52], *p* = 0.048). In contrast, neither burn severity (*β* = −0.17, 95% CI [−0.44, 0.10], *p* = 0.228) nor weather (*β* = −0.14, 95% CI [−0.33, 0.05], *p* = 0.151) showed any significance.

#### 3.4.4 Foraging Substrate Generalist Species

**FIGURE 11:**
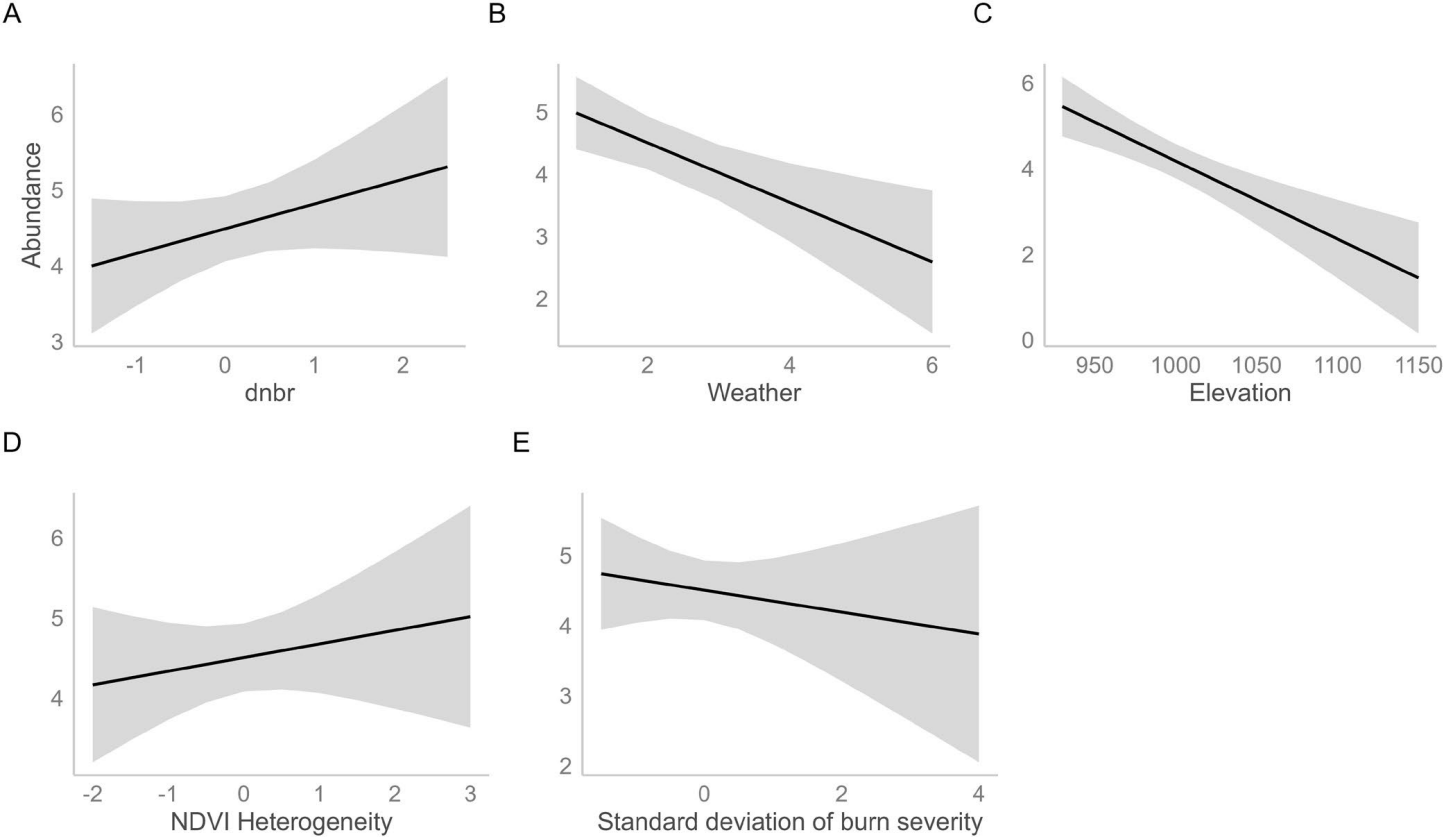
Model predicted relationships between abundance of foraging substrate generalist species and predictors: (A) burn severity (dNBR), (B) weather, (C) elevation, (D) NDVI heterogeneity, and (E) standard deviation of burn severity. Gray shaded areas denote 95% confidence intervals.

Four models were less than two delta AIC with a total weight of 0.97 (Supplementary Table S7). These four models were then averaged to identify the key predictors, revealing the abundance of foraging substrate generalists was associated with vegetation heterogeneity, burn severity (dNBR), weather, and elevation (Figures 6E and 11A–E). The averaged model explained a modest proportion of the variance in abundance of foraging substrate generalist species (R^2^ = 0.269).

Weather (*β* = −0.48, 95% CI [−0.78, −0.18], *p* = 0.002) and elevation (*β* = −0.02, 95% CI [−0.03, −0.01], *p* = < 0.001) had a significant negative relationship with abundance of foraging substrate generalists. Standard deviation of burn severity had no significant negative response (*β* = −0.32, 95% CI [−0.77, 0.13], *p* = 0.162). By contrast, burn severity (dNBR) (*β* = 0.33, 95% CI [−0.15, 0.80], *p* = 0.175) and vegetation heterogeneity (*β* = 0.32, 95% CI [−0.09, 0.73], *p* = 0.129) were positively associated with bird abundance, but not significantly.

## 4 Discussion

### 4.1 Avian Community Composition and Dominant Species

Previous studies found that, at the landscape scale, a greater diversity of fire (pyrodiversity) promoted avian diversity, while within a single fire component, diversity tended to decrease with increasing fire severity (Taylor et al. 2012; Tingley et al. 2016; Steel et al. 2019; Coelho et al. 2023). The lack of significant pairwise difference in avian species diversity across burn severity classes reinforces the importance of uniquely burned habitats and suggests the assessment of faunal diversity over a larger landscape across different wildfires. High Shannon diversity in high burn severity areas suggests disturbance may create habitat heterogeneity that supports a broader range of species, potentially opening up new niches that attract both generalist and fire‐adapted species (Wood et al. 2024). The difference observed in Shannon diversity, but not Simpson diversity, may be attributed to the sensitivity of these indices to species abundance (Magurran 2013). The dominance of Cisticolidae and Pycnonotidae in species assemblages indicates their adaptability to open and disturbed habitats, suggesting that fire may create favorable conditions for species that thrive in such environments (Aravind et al. 2010; Ramaswami et al. 2016). Furthermore, the consistent dominance of both the avian families across all burn severity classes likely reflects a pattern of biotic homogenization within the communities (Adorno et al. 2026). As generalists, they function as highly disturbance‐tolerant groups capable of capitalizing on a disturbed landscape. This pattern aligns with previous research indicating that fire disturbances often lead to postfire colonization of generalists and disturbance‐adapted species, while more specialized species may decline (Wood et al. 2024).

Our results demonstrated that avian community composition varied significantly across burn severity classes; this suggests that fire‐induced changes play a pivotal role in shaping bird assemblages in postfire landscapes (Barlow and Peres 2004; Scott and Korb 2024). The stress value of the ordination implied that our model reasonably captured the multivariate relationships of the avian community. We used the Bray‐Curtis distance measure, which was well‐suited for ecological data, especially when comparing relative species abundance between sites. The significant pairwise differences between all burn severity classes emphasize that each burn severity class has a distinct avian community. High burn severity class had the greatest divergence in species composition when compared with other burn severity classes, particularly low burn severity (Fontaine and Kennedy 2012). This was likely due to the extreme disturbance, which would attract habitat generalist species while replacing the habitat specialist species. Similarly, we found a significant difference between high and moderate severity; high and unburned areas, further highlighting that a distinct ecological niche was created by fire. Even areas with lower burn severities, such as low vs. unburned and low vs. moderate, exhibited significant differences in bird community composition. These findings suggest that even subtle changes in habitat due to fire may have a massive impact on avian assemblages. The moderate vs. unburned, while still significant, showed the smallest F‐value; this finding, while preliminary, suggests that moderate burn severity class is more similar to unburned areas in comparison to other burn severity classes. Overall, this suggests that different classes of burn severity have a profound influence on the colonization of bird species postfire. Although areas that have been burnt and those that have not often exhibited comparable levels of species richness and diversity, they commonly show pronounced dissimilarities in community composition as a result of species turnover (Barlow and Peres 2004; Beal‐Neves et al. 2020; Scott and Korb 2024). The pronounced difference in community assemblages highlights the need to understand the importance of fire heterogeneity when managing postfire landscapes as well as for prescribed fire (patch burning). However, a recent study on mixed conifer forest in Southwest Colorado also observed significantly divergent avian communities between burn severities 5 years postfire (Scott and Korb 2024). Moreover, our results emphasize that conservation strategies should account for the mosaic of burn severity, which provides distinctive ecological niches for different species to occupy. Various invasive species such as *Lantana camara* and *Senna spectabilis* have spatially spread over a large portion of the study area (Mungi et al. 2020; Anoop et al. 2025). While the effects of fire on the spread of invasive plant species in deciduous forests are known from several studies (Sukumar et al. 2005; Kodandapani et al. 2008; Verma and Jayakumar 2015), it is certain that dry season forest fires in BTR increase vegetation heterogeneity (Suresh et al. 2016), Therefore, the differences in community composition observed between burn severity classes are largely attributed to fire‐mediated effects on invasive species dynamics, although not formally incorporated into our models, as Puyravaud et al. (1995) reported that less fire‐prone areas had high tree density and high fire‐prone areas had low tree density in BTR.

### 4.2 Impact of Burn Severity on Foraging Substrate Users

In foraging substrate category, ground foraging species were significantly abundant in high burn severity area compared with unburned and low burn severity area which may be attributed to the openness and altered vegetation structure of the high burn severity areas (Raphael et al. 1987; Knaggs et al. 2020; Greenberg et al. 2023). Contrasting to the previous study, shrub‐lower canopy substrate utilizing species were significantly higher in unburned area compared with high burn severity (Smucker et al. 2005). The positive yet nonsignificant association of the abundance of foraging substrate generalist species such as *Prinia hodgsonii, Pycnonotus cafer*, and *Pycnonotus luteolus* with vegetation heterogeneity and dNBR implies that the following guilds could benefit from foraging in disturbed postfire environments (Knaggs et al. 2020; Jordan and Rachman 2024). In foraging technique category, probing species (sunbirds) were significantly higher in unburned area than that of high burn severity. This could be possibly due to higher native flowering plants in the unburned area. In addition to that we also observed relatively higher number of sunbirds and white‐eyes in the unburned and low burn severity area.

### 4.3 Bird Abundance and Its Drivers

The positive relationship between burn severity (dNBR) and bird abundance suggests that areas with higher dNBR values tend to have higher bird abundance. Jones and Tingley (2022), in their review, found that the support for the pyrodiversity‐biodiversity hypothesis for birds was equivocal. Yet this study supports the pyrodiversity‐biodiversity hypothesis while using burn severity as a single‐fire component of pyrodiversity, which also corroborates with a high positive response in forest/woodland habitat. Although the R‐squared value indicates that burn severity explains a modest proportion of the variance in abundance, it is yet statistically significant. Studies have found that avian abundance can be higher in postfire landscapes because different species exploit the variety of successional stages (Fontaine and Kennedy 2012). Higher abundance or diversity per se does not inherently indicate a functionally intact ecosystem. As species richness increases, interspecific competition for limited resources intensifies, which could be potentially disadvantageous to the native specialist species. Consequently, changes in abundance and diversity should be interpreted alongside community composition to accurately assess habitat quality.

Similarly, the positive association between vegetation heterogeneity (standard deviation of NDVI) and bird abundance strongly suggests that vegetation structural diversity enhances bird species richness and abundance by providing a wider array of ecological niches. Areas with high NDVI heterogeneity, characterized by varying vegetation density and physiological status, offer diverse foraging opportunities and habitat requirements for different bird species (Bergen et al. 2007; Price et al. 2013; Nieto et al. 2015).

In contrast, the negative effect of weather on bird abundance is reported in past literature (Walther and Pirsig 2017; Mainwaring et al. 2021). Based on the weather events, bird activity was reduced resulting in fewer detections. Bird detections were high during sunny weather and decreased when windy. The negative relationship between heterogeneity in burn severity (standard deviation of burn severity) and bird abundance presents an interesting contrast to the typical pyrodiversity findings. While studies report positive effects of pyrodiversity on bird communities (Tingley et al. 2016), the negative effect observed here may reflect several mechanisms. High spatial heterogeneity in burn severity can create fragmented habitats that may be less suitable for some bird species, particularly those requiring larger territories or specific habitat continuity. Additionally, extreme variations in burn severity within local areas might reduce habitat quality by creating abrupt transitions that are less favorable than more gradual habitat gradients.

### 4.4 Abundance of Foraging Substrate Guild and Its Drivers

Varied response of burn severity (dNBR) among foraging substrate guild, particularly with ground‐foraging species, showed a strong positive response to burn severity, whereas shrub‐lower canopy foraging species exhibited a significant negative response. Upper canopy and generalist species had a nonsignificant negative and positive response, respectively. This reflects fundamental differences in habitat requirements and foraging substrate availability following fire disturbance (Zlonis et al. 2019; Roberts et al. 2021). Ground‐foraging species benefit from the increased accessibility and prey availability that high severity burns provide through removal of dense vegetation and litter layers (Beal‐Neves et al. 2020). The exposed soil surface and reduced structural complexity facilitate foraging activities, particularly for species that hunt insects, seeds, and small invertebrates on the ground. In contrast, shrub‐lower canopy foraging species depend on intact understory vegetation, making them particularly vulnerable to complete vegetation removal in high severity areas (Zlonis et al. 2019). Upper canopy foraging species showed no significant response to burn severity; this may be attributed to the fact that forest fires in the study area are entirely surface fires while canopy fires are occasional (Suresh et al. 2016).

Vegetation heterogeneity (standard deviation of NDVI) emerged as a significant positive predictor only for upper canopy foraging species. The importance of vegetation heterogeneity for upper canopy species aligns with research demonstrating that vertical stratification and canopy complexity are critical determinants of bird community structure (Acharya and Vijayan 2017). The lack of vegetation heterogeneity effects in ground and shrub‐lower canopy guilds indicates that these groups may be more dependent on specific structural attributes rather than overall habitat heterogeneity.

Corroborating with the overall species abundance, weather showed a similar negative pattern in all foraging substrate guilds (Walther and Pirsig 2017; Mainwaring et al. 2021). Shrub‐lower canopy species were most sensitive to weather conditions, followed by ground‐foraging species, while upper canopy species showed the weakest response. Elevation showed a varied significant response for shrub‐lower canopy and generalist foraging species, suggesting that postfire recovery varies with elevation, which may explain this pattern. The absence of elevation effects for all birds may be because the point‐counts were conducted within a narrow elevational range.

## 5 Caveats

This study has some caveats as it considered a single‐fire component of the pyrodiversity framework. Firstly, we did not account for the variation in detectability across burn severity classes, which could confound the abundance estimates used in our analysis. For instance, detection probability may be systematically lower in high severity open burns where birds are more visible and audible than in dense unburned vegetation, potentially inflating apparent abundance differences. Secondly, all point counts were sampled only once and we were unable to conduct multiple temporal replicates. Single survey sampling has been suggested when inter‐point distances are less than 250 m mainly to avoid double counting of individuals. The 100 m inter‐point count stations applied in this study were due to logistic difficulties associated with the terrain, which presents a limitation regarding the independence of adjacent sampling units. While Moran's I tests did not indicate significant spatial autocorrelation for abundance or diversity, the potential for overlapping detection zones remains. Results should therefore be interpreted with the understanding that fine‐scale spatial dependence may influence the detection of highly mobile species. Studies have reported that such small inter‐point distances can substantially reduce the sample size of bird species detected and underestimate the avian richness (Morrison and Peitz 2020). In addition, single‐visit protocols can preclude estimation of detection probabilities, which are known to vary with burn severity. Consequently, observed differences in species richness and abundance between burn severity classes may partially reflect differences in detection probability rather than true ecological differences. Lastly, we could not collect systematic vegetation data at point count stations, which is a significant limitation of the study. Future studies must take multiple attributes of fire regime considering both visible and invisible mosaic of fire history; using multiple model taxa to understand the nuances of how it influences faunal species.

## 6 Conservation Implications

The 2019 forest fire burnt across different forest types creating heterogeneity in the landscape which also experienced alterations by removal of invasive species and control fire lines inside the reserve. Greater abundance per se is not ideal as different ecosystems harbor a native speciose due to habitat selection which could be altered by disturbance. The distinct avian communities associated with each burn severity class underscore the need for management strategies that promote and maintain mixed‐severity fire across the landscape. Development of mitigation strategies for the reduction of high severity burns is required as it tends to change the forest communities to more shrubland and grassland type. Management measures that promote habitat heterogeneity are likely to promote a greater diversity of species assemblages (Tingley et al. 2016; Cannon et al. 2020) like prescribed fire which promotes heterogeneity as well as reduces high severity fires. Further research should explore the specific habitat features within each burn severity category that drove these differences in community composition and assess the long‐term trajectories of bird populations postfire. Additionally, investigating species level responses, particularly for key indicator species, could provide further insights into the mechanisms driving the observed patterns. Assessing the drivers of pyrodiversity at a continental scale (Hempson et al. 2018) could help us understand the large‐scale pattern and their underlying processes. A key area of future research is to identify optimal levels of pyrodiversity for different ecosystems and taxa of conservation concern (Kelly and Brotons 2017), particularly in Asia and tropical forests that have thus far seen little pyrodiversity research (Jones and Tingley 2022; Steel et al. 2024).

## Author Contributions

**Rajagopal James Pradeeshwar:** conceptualization (equal), data curation (lead), formal analysis (lead), investigation (lead), methodology (lead), software (equal), validation (equal), visualization (equal), writing – original draft (lead), writing – review and editing (equal). **Kumar Parthasarathy Ramesh:** funding acquisition (equal), resources (supporting), supervision (supporting), writing – review and editing (supporting). **Tharmalingam Ramesh:** conceptualization (supporting), methodology (supporting), resources (supporting), supervision (supporting), writing – review and editing (supporting). **Riddhika Kalle:** conceptualization (equal), formal analysis (supporting), funding acquisition (supporting), methodology (supporting), project administration (supporting), resources (supporting), software (equal), supervision (lead), validation (supporting), visualization (supporting), writing – review and editing (supporting).

## Funding

This research was funded by the Ministry of Environment, Forest and Climate Change, Government of India (MoEFCC OM F No 19‐2/2020‐CoE‐Part (1) dated 14.12.2022) and Sálim Ali Centre for Ornithology and Natural History.

## Conflicts of Interest

The authors declare no conflicts of interest.

## Supporting information


**Figure S1:** Rarefaction curves for species diversity across four habitats (H, L, M, UB) at three diversity orders (*q* = 0, 1, 2) with increasing sample size. Curves represent diversity accumulation for species richness (*q* = 0), Shannon diversity (*q* = 1), and Simpson diversity (*q* = 2) with shaded regions showing 95% confidence intervals.
**Figure S2:** Pearson's correlation matrix showing relationships between avian community metrics and environmental variables across 120 sampling sites. Color intensity reflects correlation strength, with green for positive and orange for negative correlations.
**Figure S3:** Percent composition of (A) feeding substrate and (B) foraging technique by burn severity (UB, L, M, and H) within the 2019 forest fire, 5 years postfire.
**Table S1:**: dNBR value at the grid‐level showing the mean and standard deviation.
**Table S2: (A)** List of bird species excluded from further analysis due to low detections.
**Table S2: (B)** Total bird abundance of species across four burn severities within the 2019 forest fire, 5 years postburn.
**Table S3:** Model selection results for candidate models assessing the effects of fire and environmental variables on the total bird abundance.
**Table S4:** Model selection results for candidate models assessing the effects of fire and environmental variables on the abundance of shrub‐lower canopy foraging species.
**Table S5:** Model selection results for candidate models assessing the effects of fire and environmental variables on the abundance of ground foraging species.
**Table S6:** Model selection results for candidate models assessing the effects of fire and environmental variables on the abundance of upper canopy foraging species.
**Table S7:** Model selection results for candidate models assessing the effects of fire and environmental variables on the abundance of foraging substrate generalist species.

## Data Availability

All data supporting the findings of this study are available on Github at this link https://github.com/pradeeshwarrj/Birds_of_the_Pyrocene.git.
