# Supplementary material for: Influence of Burn Severity on Avifaunal Communities in Southern Tropical Dry Deciduous Forest, Western Ghats
Source: Ecol Evol. 2026 Aug 19;16(8):e74098. doi: 10.1002/ece3.74098 (PMC13488368; doi:10.1002/ece3.74098)
Supplement: Supplementary file 1 [file ECE3-16-e74098-s001.docx]

**Supplementary material**

**Influence of burn severity on avifaunal communities in southern tropical dry deciduous forest, Western Ghats**

**Figure S1:**  Rarefaction curves for species diversity across four habitats (H, L, M, UB) at three diversity orders (q = 0, 1, 2) with increasing sample size. Curves represent diversity accumulation for species richness (q = 0), Shannon diversity (q = 1), and Simpson diversity (q = 2) with shaded regions showing 95% confidence intervals.

**Figure S2.** Pearson’s correlation matrix showing relationships between avian community metrics and environmental variables across 120 sampling sites. Colour intensity reflects correlation strength, with green for positive and orange for negative correlations.

**Figure S3:** Percent composition of A) feeding substrate and B) foraging technique by burn severity (UB, L, M, and H) within the 2019 forest fire, five years post-fire.

**Table S1**: dNBR value at the grid-level showing the mean and standard deviation

| **Point ID** | **Burn Severity** | **Mean** | **Stdev** |
| --- | --- | --- | --- |
| H01 | High | 1.091396 | 0.020355 |
| H02 | High | 1.069715 | 0.036478 |
| H03 | High | 1.040812 | 0.073023 |
| H04 | High | 0.784295 | 0.06237 |
| H05 | High | 0.868288 | 0.075459 |
| H06 | High | 0.942691 | 0.0596 |
| H07 | High | 1.041515 | 0.033037 |
| H08 | High | 0.98351 | 0.040234 |
| H09 | High | 0.817599 | 0.100145 |
| H10 | High | 0.872114 | 0.038299 |
| H11 | High | 0.888189 | 0.033704 |
| H12 | High | 0.971779 | 0.042056 |
| H13 | High | 0.959568 | 0.049497 |
| H14 | High | 0.780706 | 0.0564 |
| H15 | High | 1.033621 | 0.032519 |
| H16 | High | 1.101293 | 0.015249 |
| H17 | High | 1.055398 | 0.030554 |
| H18 | High | 1.054373 | 0.029796 |
| H19 | High | 1.057402 | 0.032322 |
| H20 | High | 1.080489 | 0.030897 |
| H21 | High | 0.94888 | 0.069499 |
| H22 | High | 0.834798 | 0.103159 |
| H23 | High | 0.862029 | 0.083037 |
| H24 | High | 0.799933 | 0.040402 |
| H25 | High | 0.757489 | 0.050768 |
| H26 | High | 0.770112 | 0.052592 |
| H27 | High | 0.867408 | 0.065048 |
| H28 | High | 0.765221 | 0.044075 |
| H29 | High | 0.880955 | 0.06926 |
| H30 | High | 0.799038 | 0.059888 |
| L01 | High | 0.313303 | 0.028998 |
| L02 | Low | 0.340364 | 0.039381 |
| L03 | Low | 0.37429 | 0.036568 |
| L04 | Low | 0.299256 | 0.017473 |
| L05 | Low | 0.295596 | 0.016441 |
| L06 | Low | 0.317281 | 0.027005 |
| L07 | Low | 0.286917 | 0.009328 |
| L08 | Low | 0.304886 | 0.015021 |
| L09 | Low | 0.319582 | 0.015103 |
| L10 | Low | 0.290118 | 0.021874 |
| L11 | Low | 0.332038 | 0.042454 |
| L12 | Low | 0.297747 | 0.026049 |
| L13 | Low | 0.29259 | 0.011113 |
| L14 | Low | 0.26367 | 0.033045 |
| L15 | Low | 0.323407 | 0.039024 |
| L16 | Low | 0.298027 | 0.02725 |
| L17 | Low | 0.287657 | 0.029793 |
| L18 | Low | 0.300242 | 0.029986 |
| L19 | Low | 0.352098 | 0.044923 |
| L20 | Low | 0.344592 | 0.017975 |
| L21 | Low | 0.321433 | 0.019262 |
| L22 | Low | 0.342821 | 0.025639 |
| L23 | Low | 0.315568 | 0.03597 |
| L24 | Low | 0.393331 | 0.021413 |
| L25 | Low | 0.34423 | 0.017493 |
| L26 | Low | 0.389711 | 0.028559 |
| L27 | Low | 0.365417 | 0.039693 |
| L28 | Low | 0.415528 | 0.013315 |
| L29 | Low | 0.408836 | 0.012018 |
| L30 | Low | 0.410235 | 0.030258 |
| M01 | Moderate | 0.454833 | 0.057337 |
| M02 | Moderate | 0.496998 | 0.03552 |
| M03 | Moderate | 0.498294 | 0.038655 |
| M04 | Moderate | 0.522475 | 0.03796 |
| M05 | Moderate | 0.470054 | 0.0303 |
| M06 | Moderate | 0.501549 | 0.043205 |
| M07 | Moderate | 0.534832 | 0.021791 |
| M08 | Moderate | 0.555238 | 0.040463 |
| M09 | Moderate | 0.578766 | 0.035581 |
| M10 | Moderate | 0.568558 | 0.028022 |
| M11 | Moderate | 0.5326 | 0.029219 |
| M12 | Moderate | 0.501901 | 0.040947 |
| M13 | Moderate | 0.53871 | 0.02607 |
| M14 | Moderate | 0.608632 | 0.07898 |
| M15 | Moderate | 0.502448 | 0.026284 |
| M16 | Moderate | 0.563002 | 0.025917 |
| M17 | Moderate | 0.551116 | 0.032491 |
| M18 | Moderate | 0.485884 | 0.045401 |
| M19 | Moderate | 0.497164 | 0.019892 |
| M20 | Moderate | 0.591107 | 0.073917 |
| M21 | Moderate | 0.575321 | 0.034658 |
| M22 | Moderate | 0.582171 | 0.028262 |
| M23 | Moderate | 0.533248 | 0.036796 |
| M24 | Moderate | 0.559898 | 0.044693 |
| M25 | Moderate | 0.617266 | 0.039684 |
| M26 | Moderate | 0.560046 | 0.025 |
| M27 | Moderate | 0.560576 | 0.066603 |
| M28 | Moderate | 0.528999 | 0.070797 |
| M29 | Moderate | 0.518323 | 0.030659 |
| M30 | Moderate | 0.541174 | 0.015865 |
| U01 | Unburned | 0.235067 | 0.031582 |
| U02 | Unburned | 0.201435 | 0.055679 |
| U03 | Unburned | 0.235145 | 0.039758 |
| U04 | Unburned | 0.20107 | 0.06679 |
| U05 | Unburned | 0.209793 | 0.046428 |
| U06 | Unburned | 0.19102 | 0.035791 |
| U07 | Unburned | 0.166168 | 0.029823 |
| U08 | Unburned | 0.211876 | 0.039628 |
| U09 | Unburned | 0.194067 | 0.030037 |
| U10 | Unburned | 0.203587 | 0.029999 |
| U11 | Unburned | 0.157526 | 0.033307 |
| U12 | Unburned | 0.13173 | 0.029994 |
| U13 | Unburned | 0.128201 | 0.02105 |
| U14 | Unburned | 0.195857 | 0.030967 |
| U15 | Unburned | 0.20707 | 0.022271 |
| U16 | Unburned | 0.161071 | 0.033632 |
| U17 | Unburned | 0.217881 | 0.023814 |
| U18 | Unburned | 0.225674 | 0.023363 |
| U19 | Unburned | 0.21304 | 0.032904 |
| U20 | Unburned | 0.153502 | 0.018383 |
| U21 | Unburned | 0.144765 | 0.02464 |
| U22 | Unburned | 0.163002 | 0.017655 |
| U23 | Unburned | 0.205742 | 0.021482 |
| U24 | Unburned | 0.224503 | 0.022042 |
| U25 | Unburned | 0.214728 | 0.013282 |
| U26 | Unburned | 0.219577 | 0.024209 |
| U27 | Unburned | 0.25366 | 0.010327 |
| U28 | Unburned | 0.224388 | 0.020059 |
| U29 | Unburned | 0.197474 | 0.017308 |
| U30 | Unburned | 0.204692 | 0.02421 |

**Table S2A:** List of bird species excluded from further analysis due to low detections

| **S. No.** | **Common name** | **Scientific name** |
| --- | --- | --- |
| 1. | Asian koel | *Eudynamys scolopaceus* |
| 2. | Ashy Woodswallow | *Artamus fuscus* |
| 3. | Brown capped Pygmy Woodpecker | *Yungipicus nanus* |
| 4. | Changeable Hawk Eagle | *Nisaetus cirrhatus* |
| 5. | Indian Grey Hornbill | *Ocyceros birostris* |
| 6. | Indian Golden Oriole | *Oriolus kundoo* |
| 7. | Indian Cuckoo | *Cuculus micropterus* |
| 8. | Indian Paradise Flycatcher | *Terpsiphone paradisi* |
| 9. | Jungle Myna | *Acridotheres fuscus* |
| 10. | Large Cuckooshrike | *Coracina macei* |
| 11. | Malabar Whistling Thrush | *Myophonus horsfieldii* |
| 12. | Red wattled Lapwing | *Vanellus indicus* |

**Table S2B:** Total bird abundance of species across four burn severities within the 2019 forest fire, 5 years post-burn.

| Abbr. | Common Name | Scientific Name | Unburned | Low | Moderate | High |
| --- | --- | --- | --- | --- | --- | --- |
| AGBE | Asian Green Bee-eater | *Merops orientalis* |  |  |  | 4 |
| ASPR | Ashy Prinia | *Prinia socialis* | 1 | 1 |  | 14 |
| BHBA | Brown Headed Barbet | *Psilopogon zeylanicus* |  |  | 3 |  |
| BHCU | Black headed cuckooshrike | *Lalage melanoptera* | 6 | 2 | 7 | 17 |
| BHOR | Black hooded Oriole | *Oriolus xanthornus* | 8 | 4 | 2 |  |
| BRST | Brahminy Starling | *Sturnia pagodarum* |  |  |  | 14 |
| CBSU | Crimson backed Sunbird | *Leptocoma minima* | 13 | 19 | 3 | 1 |
| CHCU | Common Hawk Cuckoo | *Hierococcyx varius* | 2 |  | 1 |  |
| CITI | Cinerous Tit | *Parus cinereus* |  | 1 | 2 | 1 |
| COIO | Common Iora | *Aegithina tiphia* | 10 | 15 | 5 | 5 |
| COMY | Common Myna | *Acridotheres tristis* | 2 | 5 |  |  |
| COTA | Common Tailorbird | *Orthotomus sutorius* | 33 | 37 | 41 | 38 |
| COWO | Common Woodshrike | *Tephrodornis pondicerianus* | 7 | 1 | 4 | 1 |
| CSBA | Coppersmith Barbet | *Psilopogon haemacephalus* |  |  |  | 3 |
| ECDO | Eurasian Collared Dove | *Streptopelia decaocto* |  | 5 | 6 | 15 |
| FTDC | Fork tailed Drongo-Cuckoo | *Surniculus dicruroides* | 2 | 1 | 3 |  |
| GBCU | Grey bellied Cuckoo | *Cacomantis passerinus* | 4 | 1 | 9 | 16 |
| GBPR | Grey breasted Prinia | *Prinia hodgsonii* | 34 | 18 | 31 | 61 |
| GRJU | Grey Junglefowl | *Gallus sonneratii* | 8 | 1 | 7 | 3 |
| INPE | Indian Peafowl | *Pavo cristatus* | 3 | 1 | 5 | 1 |
| INRO | Indian Robin | *Copsychus fulicatus* | 1 |  |  | 2 |
| INWH | Indian White-eye | *Zosterops palpebrosus* | 32 | 5 | 17 | 11 |
| ISBA | Indian Scimitar Babbler | *Pomatorhinus horsfieldii* | 3 | 8 | 3 | 1 |
| JBQU | Jungle Bushquail | *Perdicula asiatica* |  |  | 3 |  |
| JUBA | Jungle Babbler | *Argya striata* | 7 |  | 11 | 15 |
| JUPR | Jungle Prinia | *Prinia sylvatica* | 28 | 21 | 28 | 28 |
| LADO | Laughing Dove | *Spilopelia senegalensis* |  | 1 | 3 | 10 |
| LECO | Lesser Coucal | *Centropus bengalensis* |  |  |  | 2 |
| LOSU | Loten's Sunbird | *Cinnyris lotenius* | 7 |  |  |  |
| LTSH | Long tailed Shrike | *Lanius schach* |  |  | 1 | 3 |
| MAPA | Malabar Parakeet | *Psittacula columboides* | 5 |  |  |  |
| OMRO | Oriental Magpie Robin | *Copsychus saularis* | 1 |  | 1 | 1 |
| PBFL | Pale billed Flowerpecker | *Dicaeum erythrorhynchos* | 7 | 7 | 10 | 2 |
| PHPA | Plum headed Parakeet | *Psittacula cyanocephala* |  |  | 2 | 3 |
| PRSU | Purple rumped Sunbird | *Leptocoma zeylonica* | 22 | 7 | 9 | 5 |
| PTBA | Puff throated Babbler | *Pellorneum ruficeps* | 9 | 12 | 12 | 4 |
| PUSU | Purple Sunbird | *Cinnyris asiaticus* | 13 | 8 | 6 | 17 |
| RRPA | Rose ringed Parakeet | *Psittacula krameri* | 1 | 3 | 4 | 3 |
| RUTR | Rufous Treepie | *Dendrocitta vagabunda* | 2 | 7 | 5 | 2 |
| RVBU | Red vented Bulbul | *Pycnonotus cafer* | 25 | 24 | 23 | 41 |
| RWBU | Red whiskered Bulbul | *Pycnonotus jocosus* | 20 | 39 | 18 | 33 |
| SBFA | Spot breasted Fantail | *Rhipidura albogularis* | 1 | 4 | 1 | 1 |
| SHIK | Shikra | *Tachyspiza badia* |  |  | 1 | 1 |
| SMMI | Small Minivet | *Pericrocotus cinnamomeus* | 7 | 7 | 1 | 2 |
| SPDO | Spotted Dove | *Spilopelia chinensis* | 5 | 8 | 4 | 13 |
| STWO | Streak throated Woodpecker | *Picus xanthopygaeus* | 1 |  |  | 3 |
| TBBA | Tawny bellied Babbler | *Dumetia hyperythra* | 2 | 2 | 6 |  |
| TBFL | Tickell's blue Flycatcher | *Cyornis tickelliae* | 7 | 12 | 13 | 8 |
| VHPA | Vernal Hanging Parrot | *Loriculus vernalis* | 4 | 11 |  |  |
| WBBU | White browed Bulbul | *Pycnonotus luteolus* | 24 | 55 | 57 | 44 |
| WBDR | White bellied Drongo | *Dicrurus caerulescens* | 5 | 1 | 1 | 4 |
| WBFA | White browed Fantail | *Rhipidura aureola* | 2 |  |  |  |
| WCBA | White cheeked Barbet | *Psilopogon viridis* | 1 | 2 | 2 | 10 |
| WTKI | White-throated Kingfisher | *Halcyon smyrnensis* | 2 |  | 1 |  |
| YBBA | Yellow billed Babbler | *Argya affinis* | 16 | 5 | 11 | 10 |
| YEBA | Yellow eyed Babbler | *Chrysomma sinense* |  |  | 1 | 5 |
|  |  | ***Abundance*** | 393 | 361 | 384 | 478 |
|  |  | ***Richness*** | 43 | 37 | 44 | 44 |
|  |  | ***Mean Abundance (±SD)*** | 11.6  (***±*** 6) | 11.1 (±4.5) | 12.1  (±4.5) | 15.2 (±4.7) |

**Table S3:** Model selection results for candidate models assessing the effects of fire and environmental variables on the total bird abundance.

|  | (Intercept) | Aspect | dnbr | Elevation | Slope | stdev_ndvi_grid | stdev_sev | Visibility | Weather | df | logLik | AICc | delta | weight |
| --- | --- | --- | --- | --- | --- | --- | --- | --- | --- | --- | --- | --- | --- | --- |
| model13 | 16.161 | NA | 1.536 | NA | NA | 1.369 | -0.980 | NA | -1.104 | 6 | -350.625 | 713.994 | 0.000 | 0.652 |
| model11 | 16.022 | NA | 1.093 | NA | NA | 1.082 | NA | NA | -1.045 | 5 | -352.652 | 715.831 | 1.836 | 0.260 |
| model10 | 15.921 | NA | 0.920 | NA | NA | NA | NA | NA | -1.002 | 4 | -355.787 | 719.921 | 5.927 | 0.034 |
| model12 | 15.979 | NA | 1.124 | NA | NA | NA | -0.504 | NA | -1.027 | 5 | -355.227 | 720.981 | 6.986 | 0.020 |
| Global | 21.558 | -0.094 | 1.419 | -0.005 | -0.505 | 1.265 | -1.017 | -0.101 | -1.170 | 10 | -349.484 | 720.987 | 6.993 | 0.020 |
| model3 | 16.462 | NA | NA | NA | NA | NA | NA | NA | -1.233 | 3 | -357.746 | 721.700 | 7.705 | 0.014 |
| model1 | 13.575 | NA | 1.434 | NA | NA | NA | NA | NA | NA | 3 | -360.426 | 727.059 | 13.065 | 0.001 |
| model2 | 15.447 | NA | NA | NA | NA | NA | NA | -0.977 | NA | 3 | -363.803 | 733.813 | 19.818 | 0.000 |
| model8 | 13.575 | NA | NA | NA | NA | 0.715 | NA | NA | NA | 3 | -364.170 | 734.548 | 20.553 | 0.000 |
| model4 | 26.220 | NA | NA | -0.013 | NA | NA | NA | NA | NA | 3 | -364.451 | 735.109 | 21.114 | 0.000 |
| model5 | 13.575 | NA | NA | NA | -0.464 | NA | NA | NA | NA | 3 | -364.862 | 735.931 | 21.936 | 0.000 |
| model7 | 13.575 | NA | NA | NA | NA | NA | 0.296 | NA | NA | 3 | -365.158 | 736.523 | 22.528 | 0.000 |
| model6 | 13.575 | -0.127 | NA | NA | NA | NA | NA | NA | NA | 3 | -365.322 | 736.851 | 22.857 | 0.000 |

**Table S4:** Model selection results for candidate models assessing the effects of fire and environmental variables on the abundance of shrub-lower canopy foraging species.

|  | (Intercept) | Aspect | dnbr | Elevation | Slope | stdev_ndvi_grid | stdev_sev | Visibility | Weather | df | logLik | AICc | delta | weight |
| --- | --- | --- | --- | --- | --- | --- | --- | --- | --- | --- | --- | --- | --- | --- |
| model14 | -6.66904 | NA | -0.66214 | 0.010132 | NA | NA | NA | NA | -0.2951 | 5 | -223.653 | 457.8889 | 0 | 0.649749 |
| model13 | -7.94073 | NA | -0.51344 | 0.010715 | NA | NA | NA | NA | NA | 4 | -225.929 | 460.2426 | 2.353741 | 0.20028 |
| model11 | 3.565747 | NA | -0.71559 | NA | NA | 0.286695 | NA | NA | -0.33835 | 5 | -226.276 | 463.1342 | 5.245364 | 0.047177 |
| model10 | 3.542032 | NA | -0.77032 | NA | NA | NA | NA | NA | -0.32336 | 4 | -227.39 | 463.1647 | 5.27584 | 0.046463 |
| model12 | 3.534461 | NA | -0.79653 | NA | NA | NA | 0.076911 | NA | -0.31855 | 5 | -227.331 | 465.2439 | 7.355021 | 0.016429 |
| model4 | -9.84169 | NA | NA | 0.012621 | NA | NA | NA | NA | NA | 3 | -229.616 | 465.4615 | 7.57263 | 0.014736 |
| Global | -8.2107 | 0.152707 | -0.59967 | 0.012103 | -0.09582 | 0.280361 | -0.15195 | -0.21451 | -0.31643 | 10 | -221.685 | 465.6149 | 7.726001 | 0.013648 |
| model1 | 2.788119 | NA | -0.61334 | NA | NA | NA | NA | NA | NA | 3 | -229.949 | 466.1264 | 8.237525 | 0.010568 |
| model8 | 2.782001 | NA | NA | NA | NA | 0.378237 | NA | NA | NA | 3 | -233.157 | 472.5425 | 14.65362 | 0.000427 |
| model2 | 2.279081 | NA | NA | NA | NA | NA | NA | 0.276001 | NA | 3 | -234.27 | 474.7692 | 16.88033 | 0.00014 |
| model3 | 3.106562 | NA | NA | NA | NA | NA | NA | NA | -0.13182 | 3 | -234.518 | 475.2644 | 17.37554 | 0.00011 |
| model7 | 2.789159 | NA | NA | NA | NA | NA | -0.16836 | NA | NA | 3 | -234.65 | 475.5293 | 17.64045 | 9.60E-05 |
| model6 | 2.797364 | 0.147726 | NA | NA | NA | NA | NA | NA | NA | 3 | -234.685 | 475.5991 | 17.71024 | 9.27E-05 |
| model5 | 2.799293 | NA | NA | NA | 0.121141 | NA | NA | NA | NA | 3 | -234.784 | 475.7959 | 17.90705 | 8.40E-05 |

**Table S5:** Model selection results for candidate models assessing the effects of fire and environmental variables on the abundance of ground foraging species.

|  | (Intercept) | Aspect | dnbr | Elevation | Slope | stdev_ndvi_grid | stdev_sev | Visibility | Weather | df | logLik | AICc | delta | weight |
| --- | --- | --- | --- | --- | --- | --- | --- | --- | --- | --- | --- | --- | --- | --- |
| model1 | 5.794096 | NA | 1.274376 | NA | NA | NA | NA | NA | NA | 3 | -285.103 | 576.4144 | 0 | 0.399201 |
| model10 | 6.337781 | NA | 1.153472 | NA | NA | NA | NA | NA | -0.23138 | 4 | -284.298 | 576.9464 | 0.531953 | 0.305971 |
| model11 | 6.378567 | NA | 1.284002 | NA | NA | NA | -0.32732 | NA | -0.2482 | 5 | -283.553 | 577.6364 | 1.221944 | 0.216696 |
| model12 | 6.386064 | NA | 1.305464 | NA | NA | 0.068556 | -0.35073 | NA | -0.2517 | 6 | -283.518 | 579.7859 | 3.371437 | 0.073976 |
| Global | 7.47697 | -0.13488 | 1.387319 | -0.00166 | -0.32281 | 0.024029 | -0.38001 | 0.358445 | -0.30487 | 10 | -281.785 | 585.6065 | 9.192066 | 0.004029 |
| model3 | 7.033423 | NA | NA | NA | NA | NA | NA | NA | -0.52322 | 3 | -293.33 | 592.8685 | 16.45402 | 0.000107 |
| model4 | 12.97726 | NA | NA | -0.00717 | NA | NA | NA | NA | NA | 3 | -296.428 | 599.0644 | 22.64994 | 4.82E-06 |
| model7 | 5.804334 | NA | NA | NA | NA | NA | 0.294783 | NA | NA | 3 | -296.703 | 599.6142 | 23.19978 | 3.66E-06 |
| model8 | 5.809667 | NA | NA | NA | NA | -0.29389 | NA | NA | NA | 3 | -296.709 | 599.6265 | 23.21204 | 3.64E-06 |
| model2 | 6.461152 | NA | NA | NA | NA | NA | NA | -0.34307 | NA | 3 | -296.738 | 599.6853 | 23.27091 | 3.53E-06 |
| model5 | 5.804208 | NA | NA | NA | -0.20082 | NA | NA | NA | NA | 3 | -297.026 | 600.2598 | 23.84541 | 2.65E-06 |
| model6 | 5.805573 | -0.18686 | NA | NA | NA | NA | NA | NA | NA | 3 | -297.059 | 600.3262 | 23.91177 | 2.56E-06 |

**Table S6:** Model selection results for candidate models assessing the effects of fire and environmental variables on the abundance of upper canopy foraging species.

|  | (Intercept) | Aspect | dnbr | Elevation | Slope | stdev_ndvi_grid | stdev_sev | Visibility | Weather | df | logLik | AICc | delta | weight |
| --- | --- | --- | --- | --- | --- | --- | --- | --- | --- | --- | --- | --- | --- | --- |
| model8 | 1.97124 | NA | NA | NA | NA | 0.275326 | NA | NA | NA | 3 | -126.442 | 259.1923 | 0 | 0.29267 |
| model10 | 1.979845 | NA | -0.13413 | NA | NA | 0.243714 | NA | NA | NA | 4 | -125.88 | 260.2792 | 1.086859 | 0.169969 |
| model11 | 2.297291 | NA | -0.19884 | NA | NA | 0.253296 | NA | NA | -0.14029 | 5 | -124.775 | 260.3404 | 1.148112 | 0.164842 |
| model1 | 1.997265 | NA | -0.18931 | NA | NA | NA | NA | NA | NA | 3 | -127.638 | 261.583 | 2.390659 | 0.088563 |
| model12 | 0.146029 | NA | -0.16447 | 0.002131 | NA | 0.2634 | NA | NA | -0.13128 | 6 | -124.433 | 261.9852 | 2.792836 | 0.072431 |
| model4 | -0.91005 | NA | NA | 0.002901 | NA | NA | NA | NA | NA | 3 | -128.127 | 262.5607 | 3.368421 | 0.054317 |
| model3 | 2.139485 | NA | NA | NA | NA | NA | NA | NA | -0.0676 | 3 | -128.494 | 263.2962 | 4.103841 | 0.037605 |
| model6 | 1.990327 | 0.059105 | NA | NA | NA | NA | NA | NA | NA | 3 | -128.659 | 263.6249 | 4.432591 | 0.031905 |
| model5 | 1.986884 | NA | NA | NA | -0.02199 | NA | NA | NA | NA | 3 | -128.751 | 263.8089 | 4.616568 | 0.029101 |
| model2 | 2.017348 | NA | NA | NA | NA | NA | NA | -0.01505 | NA | 3 | -128.758 | 263.8236 | 4.631261 | 0.028888 |
| model7 | 1.987672 | NA | NA | NA | NA | NA | 0.004853 | NA | NA | 3 | -128.763 | 263.8338 | 4.641514 | 0.02874 |
| Global | -0.7631 | 0.045569 | -0.21404 | 0.00337 | -0.05081 | 0.248701 | -0.00659 | -0.1691 | -0.12832 | 10 | -123.756 | 270.6106 | 11.41824 | 0.00097 |

**Table S7:** Model selection results for candidate models assessing the effects of fire and environmental variables on the abundance of foraging substrate generalist species.

|  | (Intercept) | Aspect | dnbr | Elevation | Slope | stdev_ndvi_grid | stdev_sev | Visibility | Weather | df | logLik | AICc | delta | weight |
| --- | --- | --- | --- | --- | --- | --- | --- | --- | --- | --- | --- | --- | --- | --- |
| model15 | 23.11767 | NA | 0.443577 | -0.01796 | NA | 0.364394 | -0.36568 | NA | -0.49438 | 7 | -233.651 | 482.3885 | 0 | 0.295243 |
| model12 | 23.46119 | NA | 0.236124 | -0.01837 | NA | NA | NA | NA | -0.46743 | 5 | -235.973 | 482.5176 | 0.129028 | 0.276798 |
| model13 | 23.37241 | NA | 0.275914 | -0.01827 | NA | 0.262606 | NA | NA | -0.47034 | 6 | -235.062 | 482.9316 | 0.543056 | 0.225039 |
| model14 | 23.31194 | NA | 0.339253 | -0.01818 | NA | NA | -0.24771 | NA | -0.48295 | 6 | -235.277 | 483.3624 | 0.973873 | 0.181429 |
| Global | 23.19725 | -0.13812 | 0.397995 | -0.0178 | -0.00871 | 0.370206 | -0.35639 | -0.13694 | -0.48611 | 10 | -233.19 | 488.5806 | 6.192105 | 0.013353 |
| model11 | 20.7428 | NA | 0.474275 | -0.01675 | NA | NA | NA | NA | NA | 4 | -240.802 | 489.9816 | 7.593085 | 0.006628 |
| model4 | 22.14209 | NA | NA | -0.01813 | NA | NA | NA | NA | NA | 3 | -243.451 | 493.126 | 10.73749 | 0.001376 |
| model10 | 4.927266 | NA | 0.400924 | NA | NA | NA | NA | NA | -0.3845 | 4 | -245.317 | 499.0118 | 16.62325 | 7.25E-05 |
| model3 | 5.175745 | NA | NA | NA | NA | NA | NA | NA | -0.48272 | 3 | -246.9 | 500.0252 | 17.63672 | 4.37E-05 |
| model1 | 4.018064 | NA | 0.587812 | NA | NA | NA | NA | NA | NA | 3 | -248.173 | 502.5704 | 20.18185 | 1.22E-05 |
| model2 | 5.196334 | NA | NA | NA | NA | NA | NA | -0.60854 | NA | 3 | -249.186 | 504.5966 | 22.20811 | 4.44E-06 |
| model5 | 4.052179 | NA | NA | NA | -0.26541 | NA | NA | NA | NA | 3 | -251.058 | 508.3402 | 25.95168 | 6.84E-07 |
| model8 | 4.047005 | NA | NA | NA | NA | 0.172164 | NA | NA | NA | 3 | -251.476 | 509.1757 | 26.7872 | 4.50E-07 |
| model6 | 4.046886 | -0.0984 | NA | NA | NA | NA | NA | NA | NA | 3 | -251.674 | 509.573 | 27.1845 | 3.69E-07 |
| model7 | 4.044754 | NA | NA | NA | NA | NA | 0.062941 | NA | NA | 3 | -251.734 | 509.6928 | 27.30427 | 3.48E-07 |
